# Estimation of groundwater storage loss using surface–subsurface hydrologic modeling in an irrigated agricultural region

**DOI:** 10.1038/s41598-025-92987-6

**Published:** 2025-03-11

**Authors:** Salam A. Abbas, Ryan T. Bailey, Jeremy T. White, Jeffrey G. Arnold, Michael J. White

**Affiliations:** 1 Department of Civil and Environmental Engineering, Colorado State University, Fort Collins, CO 80521 USA; 2 INTERA Incorporated, Fort Collins, CO 80524 USA; 3 Grassland Soil and Water Research Laboratory, USDA–ARS, Temple, TX 76502 USA

**Keywords:** Hydrology, Natural hazards

## Abstract

In the Mississippi alluvial plain (MAP) area, the demand for groundwater resources from the alluvial aquifer for agricultural irrigation has led to significant reductions in groundwater-level elevation over time. In this study, we use the hydrologic model SWAT + to quantify long-term changes in groundwater storage within the MAP in United States, wherein groundwater is used extensively for irrigation. We apply a linear quantile regression method to perform trend analysis for wet, dry, and average conditions for the 1982–2020 period. The SWAT + model uses the *gwflow* module to simulate groundwater storage and groundwater-surface water interactions in a physically based spatially distributed manner, with groundwater pumping linked to field-based irrigation demand. Results indicate significant trends in storage and groundwater fluxes. In wet conditions, significant decline trends are noted in groundwater head (–18.0 mm/yr.) and groundwater evapotranspiration (–0.7 mm/yr.). Under dry conditions, trends are in groundwater head (–28.0 mm/yr.), recharge (–5.5 mm/yr.), and groundwater discharge (–5.5 mm/yr.). For average conditions, decreases include groundwater head (–20.6 mm/yr.), recharge (–6 mm/yr.), and groundwater discharge (–9.3 mm/yr.). This underscores the significance of local management solutions.

**Supplementary Information:**

The online version contains supplementary material available at 10.1038/s41598-025-92987-6.

## Introduction

Groundwater fulfills social water demands through municipal, industrial, and agricultural purposes, and groundwater depletion may arise when extractions outpace recharge rates^1–3^. Aquifer systems are integral to global hydrological and biochemical cycles, significantly contributing to ecosystem sustainability. In recent decades, groundwater resources have been progressively utilized, leading to significant groundwater storage loss in many regions globally^4–8^. For example, during the 20th century alone the United States experienced a depletion of approximately 800 km^3^ of groundwater storage^9^.

While the effects of groundwater pumping are most pronounced at local levels, groundwater depletion constitutes a worldwide issue due to its extensive prevalence and possible ramifications for water and food security^10,11^. The ramifications of groundwater depletion are intricate and contingent upon the aquifer, yet some issues are prevalent^12^. The primary consequence is a drop in water tables. This results in increased pumping costs or depletion of wells, negatively affecting users; diminished groundwater flow to streams, springs, and wetlands, adversely affecting ecosystems; and land subsidence, which irreversibly decreases storage capacity and could damage infrastructure^8,13,14^. Excessive groundwater exploitation has a negative influence on the ecosystem and water quality^15,16^.

In regions where agriculture relies on groundwater, monitoring groundwater storage is crucial for evaluating its long-term sustainability^17–19^. Nonetheless, groundwater monitoring systems are occasionally omitted due to the substantial expenses associated with their installation and maintenance. That has resulted in increasing interest in using remote sensing data^20,21^, regional groundwater modeling^9,22^, global hydrologic models^23,24^, and machine learning^5,25^ to assess changes in groundwater storage. The assessment of groundwater storage loss is crucial for assisting managers in formulating appropriate strategies for water resource planning and management^26,27^. The loss of groundwater storage can be determined using the balance method that quantifies the pertinent input and output variables. Groundwater balances are typically assessed by empirical relationships or mathematical modeling techniques, such as GSFLOW^28,29^.

Many regional groundwater models predominantly depend on groundwater level data for calibration, potentially leading to nonunique solutions for storage and fluxes^30,31^. The use of other watershed responses such as streams and evapotranspiration, enhances the precision of model parameters and predictions. The combination of streamflow, groundwater head, and evapotranspiration serves to constrain hydrologic fluxes within realistic ranges. Most of groundwater storage loss studies used the non–parametric trend test^32,33^ to assess whether there are significant trends and to identify the possible causes of those changes^34–36^. However, the trend analysis of average or aggregated values presents limitations for regional water resources assessment, which typically requires detailed information regarding extreme scenarios, such as floods and droughts^37,38^.

Furthermore, examining trends of other hydrologic fluxes such as groundwater recharge, groundwater evapotranspiration alongside groundwater head is fundamental for identifying groundwater storage loss in intensively irrigated regions. Each flux distinctly influences the equilibrium between inflows and outflows in an aquifer, directly affecting storage levels and sustainability. By analyzing these trends collectively, researchers can identify principal factors contributing to depletion and formulate focused methods for aquifer management.

The Mississippi Alluvial Plain (MAP) has emerged as significant agricultural region in the United States, highly dependent on groundwater for irrigation^39–41^. Despite the abundance of rainfall, its timing and volume frequently fail to align with agricultural requirements. Consequently, producers have progressively adopted irrigation to enhance yields and reduce hazards linked to drought^42^. Each day, almost 12 billion gallons are extracted from the alluvial aquifer of the Mississippi River Valley^43^. The extensive utilization of available groundwater resources has led to considerable reductions in groundwater levels and base flows in streams within the alluvial plain^44–46^.

The objective of this study is to assess the long–term groundwater storage loss in the Mississippi Delta (northwest Mississippi, USA) (28,121 km²), an area characterized by substantial irrigation withdrawal volumes^47^ and significant groundwater depletion rates^48,49^. We utilize the surface–subsurface hydrologic model, the Soil and Water Assessment Tool (SWAT+)^50,51^ amended with new *gwflow* module^52,53^ to simulate physically based spatially distributed groundwater flow, storage and groundwater head with an unconfined aquifer in connection with a stream networks. The model simulates land surface hydrology, groundwater hydrology, channel hydrology, and soil hydrology on a daily basis, incorporating fluxes such as surface runoff, soil evapotranspiration, lateral soil flow, infiltration, recharge to the water table, deep percolation, groundwater interaction with streams, and groundwater interaction with lakes and reservoirs. This study connects cultivated areas allocated for groundwater irrigation to underlying aquifer grid cells, from which groundwater is taken according to irrigation demand, the efficiencies of selected irrigation methods (flood, sprinkler, drip), and the available groundwater storage. Irrigation is activated according to soil moisture levels. This process mimics daily groundwater extraction for each agricultural land inside the model domain.

The SWAT + model simulation spans the 2000–2020 period, including a two-year warm-up period (2000–2001), a seven-year calibration period (2002–2008), and a twelve-year testing period (2009–2020), using measurements of streamflow, groundwater head, and surface evapotranspiration at sites throughout the river basin. Then the calibrated SWAT + model is used to investigate long-term of simulated groundwater head, groundwater evapotranspiration, groundwater pumping, recharge, and groundwater seepage. The linear quantile regression^54,55^ is used to perform trend analysis for both extremely wet and dry conditions as well as the average condition. The model is developed utilizing a collection of national datasets, with the irrigation source for each field predicted based on county-level USGS water consumption statistics^56^. The model output is evaluated against critical system response variables that limit soil moisture and accessible groundwater storage, hence influencing irrigation demand and supply: soil evapotranspiration, streamflow, crop yield, and groundwater levels.

## Materials and methods

### Study region: Mississippi alluvial plain (MAP)

**Fig. 1:**
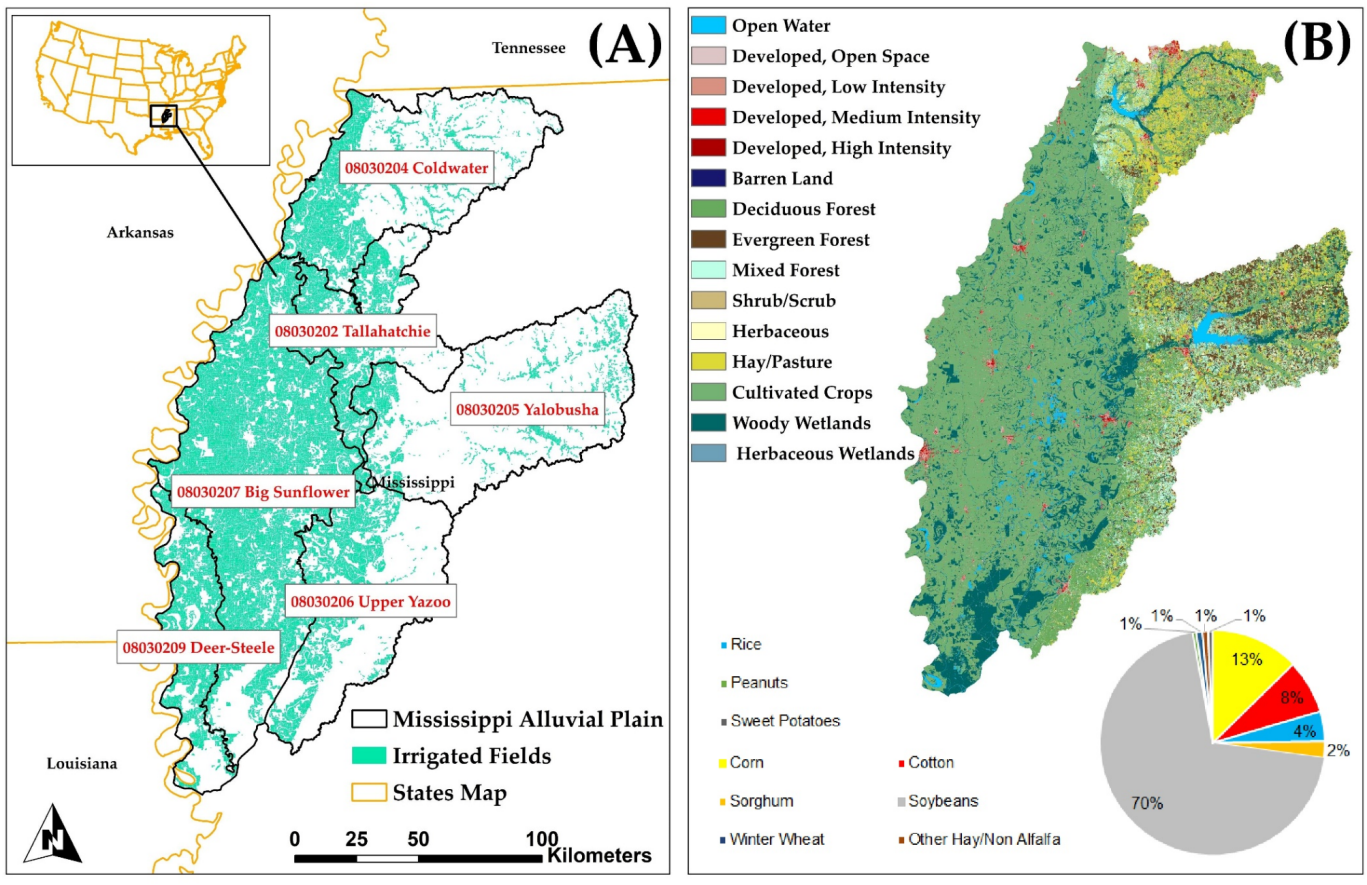
(A) Study region boundary, in northwest Mississippi, USA. Cultivated fields are shown in green. (B) land cover for the study area, displaying (pie chart) portion of cultivated land for each crop type^57^.

**Fig. 2:**
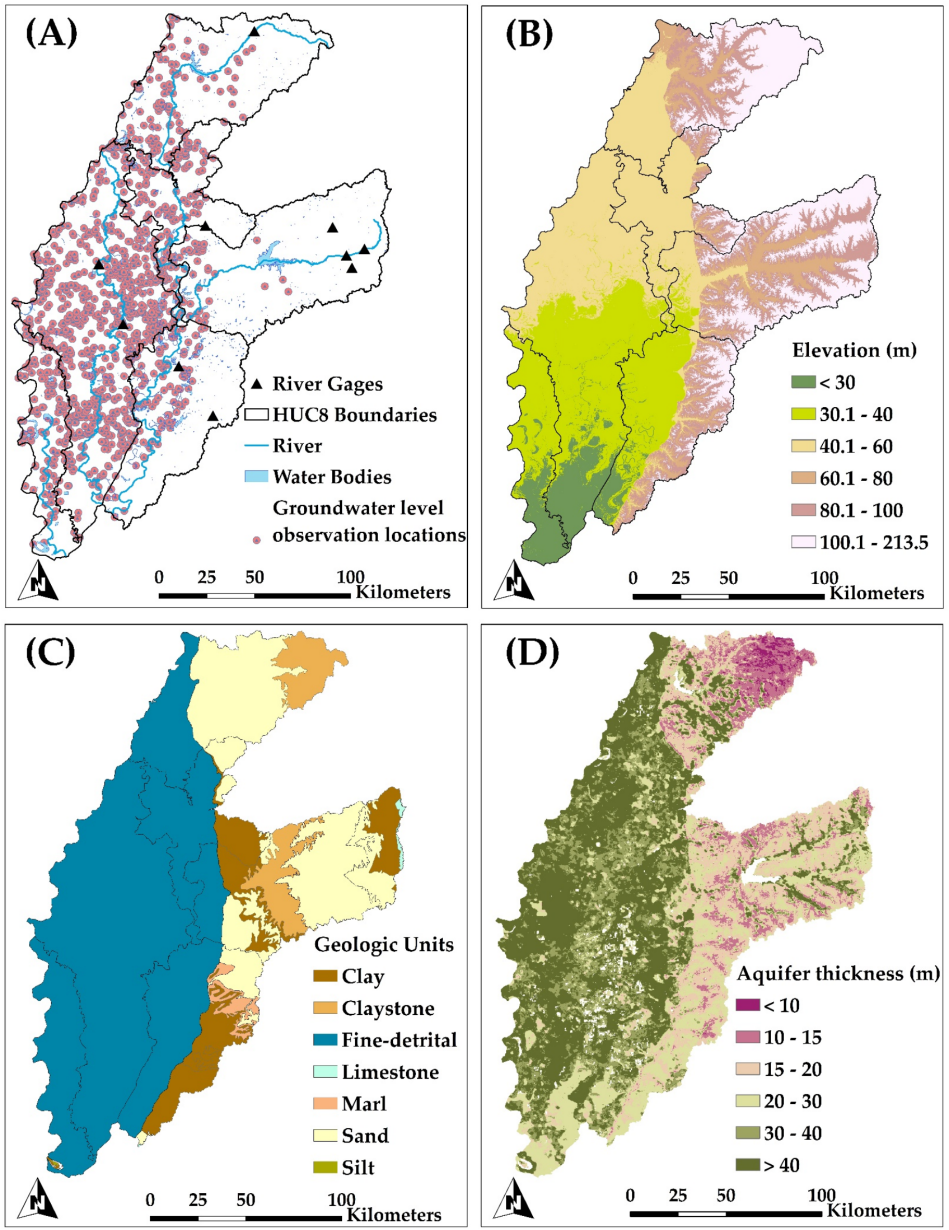
Geographic characteristics and model inputs: (A) watershed boundaries, USGS monitoring wells, water bodies, Main rivers, and USGS river gage locations; (B) topographic elevation (30-m resolution); (C) geologic units; and (D) aquifer thickness (m) (500-m resolution).

The study area encompasses six 8-digit HUC (Hydrologic Unit Code) watersheds (Fig. 1A) with elevation ranges 22–213 m (Fig. 2B). These watersheds includes the section of the Mississippi Alluvial Plain (MAP) located in northwestern Mississippi, also known as the Mississippi ‘Delta’. The watershed delineation follows the hierarchical HUC system established by the United States Geological Survey (USGS) and the Natural Resources Conservation Service (NRCS), where HUC8 delineates larger drainage areas (sub-basins), and HUC12 provides finer subdivisions within these areas. The MAP is among the most prolific agricultural areas in the United States, yielding substantial amounts of soybean, rice, cotton, and corn. The watershed areas range from 2,121 km^2^ (Deer-Steele) to 8,210 km^2^ (Big Sunflower). About 80% of the Delta’s land is used for major crops, which include rice, corn, cotton, and soybeans (Fig. 1B)^57^. Despite high average annual rainfall depths, most precipitation transpires during the non-growing season, requiring irrigation from surface water or groundwater^58^.

The predominant source of groundwater is the alluvial aquifer, characterized by a thickness ranging from 7 to 45 m (Fig. 2D) and consisting of sand, gravel, silt, and fine clay deposits (Fig. 2C) from the Quaternary period^59^, formed during historical flooding events of the Mississippi River^60^. The MAP ranks second in groundwater withdrawal quantities for irrigation in the United States, surpassed only by the High Plains Aquifer^47^.

The alluvial aquifer provides the majority of irrigation water, and Mississippi rates eighth nationally in irrigated cropland area due to irrigation in the Delta^61^. The substantial extraction of groundwater from over 18,000 wells^62^ has resulted in a fall in groundwater levels by 0.15 m to 0.45 m annually in recent decades^63^, with the most significant reductions occurring in the central Delta region^64,65^. These reductions have resulted in less baseflow to streams, especially for the Big Sunflower River (see to Fig. 1 for location). To mitigate decreasing groundwater levels, alternative water management options have been proposed and examined^66^, including enhanced irrigation efficiency, on-farm storage and tailwater recovery, and intra-basin transfer of surface water.

### SWAT + hydrologic model

The Soil and Water Assessment Tool (SWAT)^50^ is a semi-distributed, process-based, basin scale hydrologic model that has been extensively utilized for simulating hydrologic processes and water quality in watershed systems^67–69^. SWAT simulates hydrologic fluxes at a higher spatial resolution by grouping the watershed into hydrologic response units (HRUs). HRUs are computational units, each with unique slope, land use, and soil composition^70^. The SWAT + model^51^, the most recent enhancement of SWAT, provides enhanced hydrologic interconnections within a watershed system. SWAT + can simulate the transport of sediment, water, and nutrients through a network of interconnected hydrologic units (HRUs), landscape units, aquifers, ponds, wetlands, reservoirs, and channels within the watershed’s landscape. The watershed is divided into routing units that consolidate hydrographs and sediment/nutrient mass from several HRUs, enabling their movement over the landscape and along the channel network.

The present study employs SWAT + models from the National Agroecosystem Model (NAM)^71,72^, a comprehensive effort designed to evaluate national conservation initiatives. A distinct SWAT + model is developed for each of the 2,121 8-digit (HUC8) watersheds inside the NAM in the contiguous United States. This study employs model configurations applied to the 6 designated sub-watersheds (refer to Table 1). The datasets utilized for the creation of the six models are presented in Table 2. Every stream and lake segment in the NHD + dataset^73^ (Fig. 2A) constitutes a distinct channel and reservoir entity, respectively. Hydrologic Response Units (HRUs) are defined by (1) individual field boundaries^74^ (Fig. 1A) and (2) distinct geographic intersections of topographic slope (Fig. 2B), soil type, and land use (Fig. 1B).

**Fig. 3:**
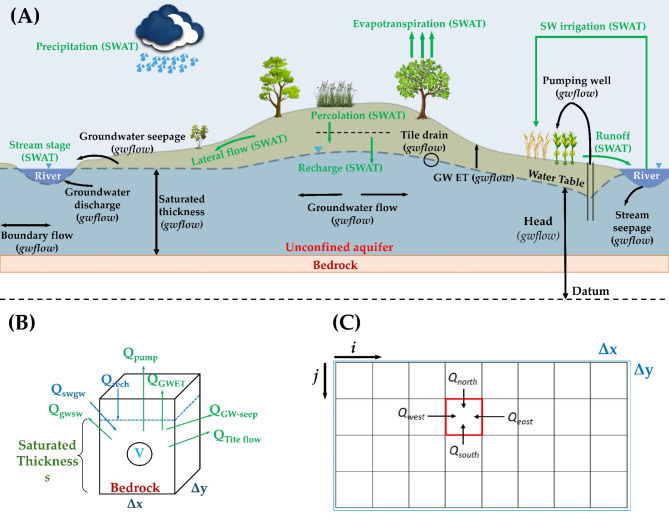
Illustrative representation of hydrologic fluxes in a typical watershed stream-aquifer system: demonstrating (A) key hydrologic components for SWAT + and gwflow; (B) the hydrologic fluxes for each individual cell; and (C) close-up of grids.

This resulted in a total of 63,078 HRUs, 16,043 channels, and 312 subbasins for the region, with each subbasin corresponding to a 12-digit catchment (Table 1). Crop rotations are supplied by USDA-NASS and CDL. Daily meteorological data are derived from a synthesis of GHCN and PRISM^72^, employing methodologies outlined in Gao et al. (2020)^75^. This study use the groundwater flow module (*gwflow*)^52^ routine of SWAT + to model groundwater processes, instead of utilizing the original aquifer module. The *gwflow* module models groundwater storage, flow, and inflows and outflows in a physically based, spatially distributed manner, utilizing a series of interconnected aquifer control volumes (i.e., cells) that encompass the spatial extent of a single-layer unconfined aquifer within a watershed^76,77^. The diagrammatic representation of hydrologic fluxes in a typical watershed stream-aquifer system is depicted in Fig. 3. The thickness of each cell corresponds to the total thickness of the aquifer, extending from the bedrock to the ground surface, with the SWAT + model simulating the vadose zone and the *gwflow* module simulating groundwater flow processes.

The cell size for the *gwflow* module was established at 500 m for each of the six watersheds (see to Table 1). The data necessary for processing *gwflow* cell values (see to Table 2) include aquifer thickness (shown in Fig. 2D), together with hydraulic conductivity (*K*) and specific yield (*S*_*y*_) sourced from geological maps (also presented in Fig. 2C).

### Demand-responsive irrigation simulation

**Fig. 4:**
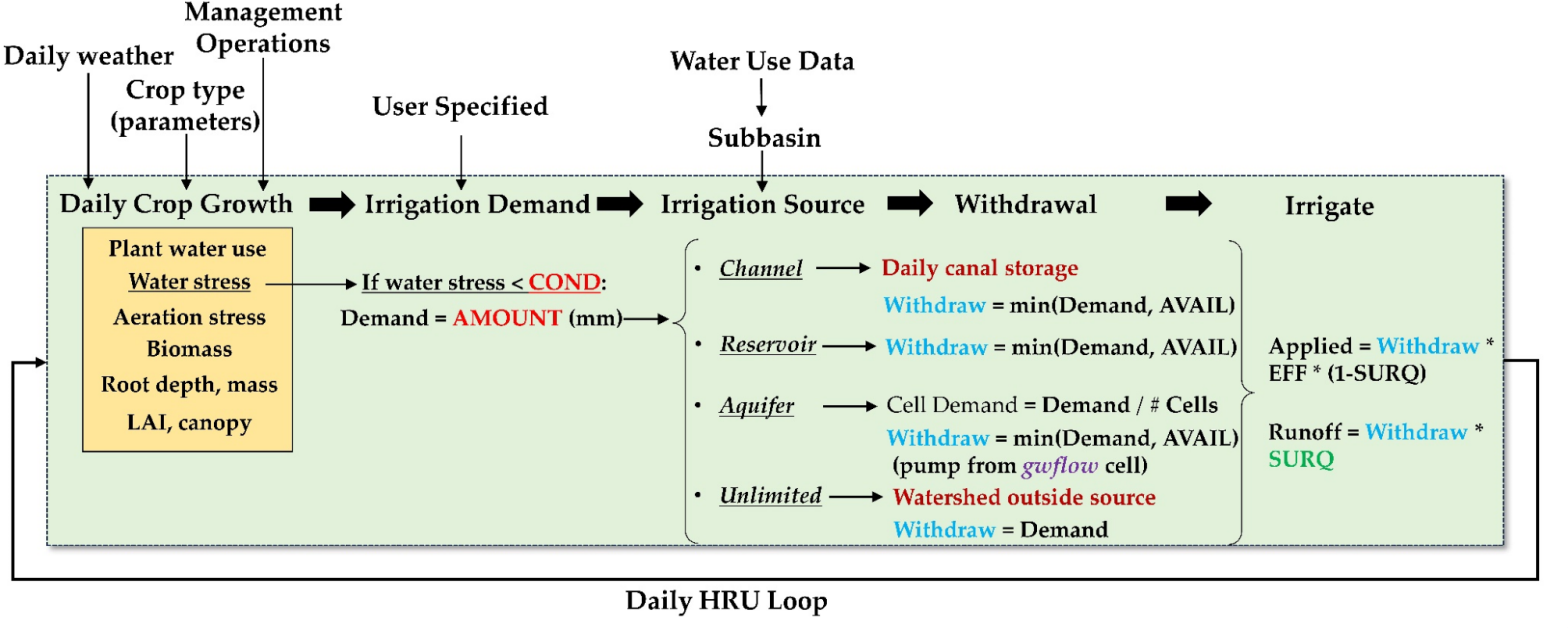
Flow process within the SWAT + code for simulating demand-driven irrigation events at each field HRU on a daily basis.

The water allocation module of SWAT+, in conjunction with decision tables, simulates irrigation demand and application. A decision table provides a series of conditions and corresponding actions that outline management processes within the simulation. The two factors for irrigation are plant growth and a soil water stress level of 0.8. The resultant action is an irrigation event, with water sourced from a designated stream channel, reservoir, or aquifer. Figure 4 represents the process by which an irrigation event is triggered for a particular HRU. In a certain Hydrologic Response Unit (HRU), crop development is affected by daily meteorological factors like precipitation, temperature, sun radiation, relative humidity, and wind speed, alongside management practices such as planting and fertilizer application.

Crop growth is limited by temperature, lack of nutrients and water stress, the latter is a function of rooting depth and soil water availability. When the soil water content attains a specified threshold (COND) indicative of water stress (the ratio of actual water uptake to the maximum potential water uptake), an irrigation event is initiated, and a request for irrigation (AMOUNT, mm) is generated. The water demand volume (m^3^) is compared to the available volume (AVAIL) in the source object (channel, reservoir, aquifer) to see whether the whole demand can be satisfied. If the demand volume exceeds AVAIL, the demand is adjusted to equal AVAIL, and the volume in the source object is reduced to 0. If the demand volume is less than AVAIL, the demand volume is deducted from the source object and utilized as irrigation water for the HRU, applying a specified irrigation efficiency (EFF) and a surface runoff ratio (SURQ).

When the *gwflow* module is active and groundwater is identified as the irrigation source, the unconfined aquifer underlying the demand HRU supplies groundwater for irrigation purposes. The demand volume is initially assessed against the available groundwater in the aquifer grid cells. If demand is less than the available storage, the total volume of demand is withdrawn from the grid cells and designated to the HRU as irrigation water. However, if demand surpasses the available storage, any excess storage is eliminated for irrigation purposes. Groundwater depletion and storage limitations are incorporated into the simulation. In the MAP model, 41,824 fields are irrigated utilizing this methodology, 20,996 fields are irrigated by groundwater.

### Model calibration and testing

In this study, we use the iterative Ensemble Smoother (iES) algorithm^78^ as implemented in PEST + +^79^ for model calibration. The iES algorithm treats the calibration analysis as a Bayesian inverse problem, where parameters are treated stochastically through the explicit use of ensemble of parameter realizations. The iES iterative process adjusts these parameter realizations until the discrepancy between historic observations and the simulated equivalents are minimized in weighted L-2 norm sense. These adjustments are made through a series of consecutive update procedures to account for nonlinearity in the relationship between parameters and model outputs.

For this study, the SWAT + models was constructed for the period 2000–2020 using the datasets presented in Table 2. The warm-up period is from 2000 to 2001, the calibration period from 2002 to 2008, and the testing period from 2009 to 2020. The model streamflow responses were evaluated using the Nash–Sutcliffe Efficiency Index (NSE), percent of bias (PBIAS), Kling–Gupta Efficiency Index (KGE), and determination coefficient (R^2^). The evaluation of simulated groundwater head is conducted utilizing mean absolute error (MAE). The model parameters used in the calibration are listed in Table 3, These parameters control specific hydrologic processes, including surface runoff, channel flow, groundwater flow and storage, potential and actual evapotranspiration, soil and water storage, and soil lateral flow. Observation data to evaluate model response to historic conditions consist of monthly streamflow (m^3^/sec) from USGS stream gages (10 locations; Fig. 2A) and annual average groundwater head (m) from USGS monitoring wells (1155 locations; Fig. 2A). The hydrologic models are also assessed using watershed average monthly actual evapotranspiration using TerraClimate model^80^.

### Trend analysis using quantile regression

Quantile Regression (QR)^54,55^ is a statistical methodology first developed for regression analysis in econometrics, serving as an alternative and potentially superior approach to the ordinary least squares method (OLS). It has since been progressively implemented across various other disciplines. The method has garnered significant attention in several statistical literatures, although it has been less emphasized in the domains of water resources analysis and environmental research^81^. Abbas and Xuan (2019)^37^ and Papacharalampous et al. (2020)^82^ provide a review of various papers that demonstrate the application of quantile regression in water and environmental research.

The QR approach is a robust enhancement of standard linear regression, wherein quantiles, rather than the mean of the response variables, are conditioned on independent variables, offering numerous advantages as detailed in Koenker (2005)^55^. The origin of QR is documented in many literatures^83–86^. Essentially, consider *Y* is a random response variable which is related to M predictors at time t, i.e. *x*_*i*_*(t) (i = 1*,* 2…*,* M)*, the generic form of the linear quantile regression for a given quantile τ of the predictand at same time t, i.e. *y(t)* can be formulated as:

1 $$\:{\widehat{y}}_{\tau\:}\left(t\right)=\sum\:_{i=1}^{M}{b}_{i}{x}_{i}\left(t\right)+{b}_{0}$$

where the intercept *b*_*0*_ and the gradients bi can be computed by minimizing the quantile error function

2 $$\:{E}_{\tau\:}=\frac{1}{N}\sum\:_{t=1}^{N}{\rho\:}_{\tau\:}\left[y\left(t\right)-{\widehat{y}}_{\tau\:}\left(t\right)\right]$$

The appropriate optimization procedures are described in detail in Koenker (2005)^55^, resulting in the linear quantile regression model. While the linear variant of QR is prevalent, parametric models that exhibit non-linearity in parameters (i.e., models where the non-linear regression equation is explicitly defined by the modeler) can also be estimated^87^. The linear representation of this relationship (Eq. 1) can be employed to characterize the magnitude (regarding its gradient) of the trend. Proposing a linear relationship between the quantiles of the response variable and the input variable (time) renders the process parametric.

**Fig. 5:**
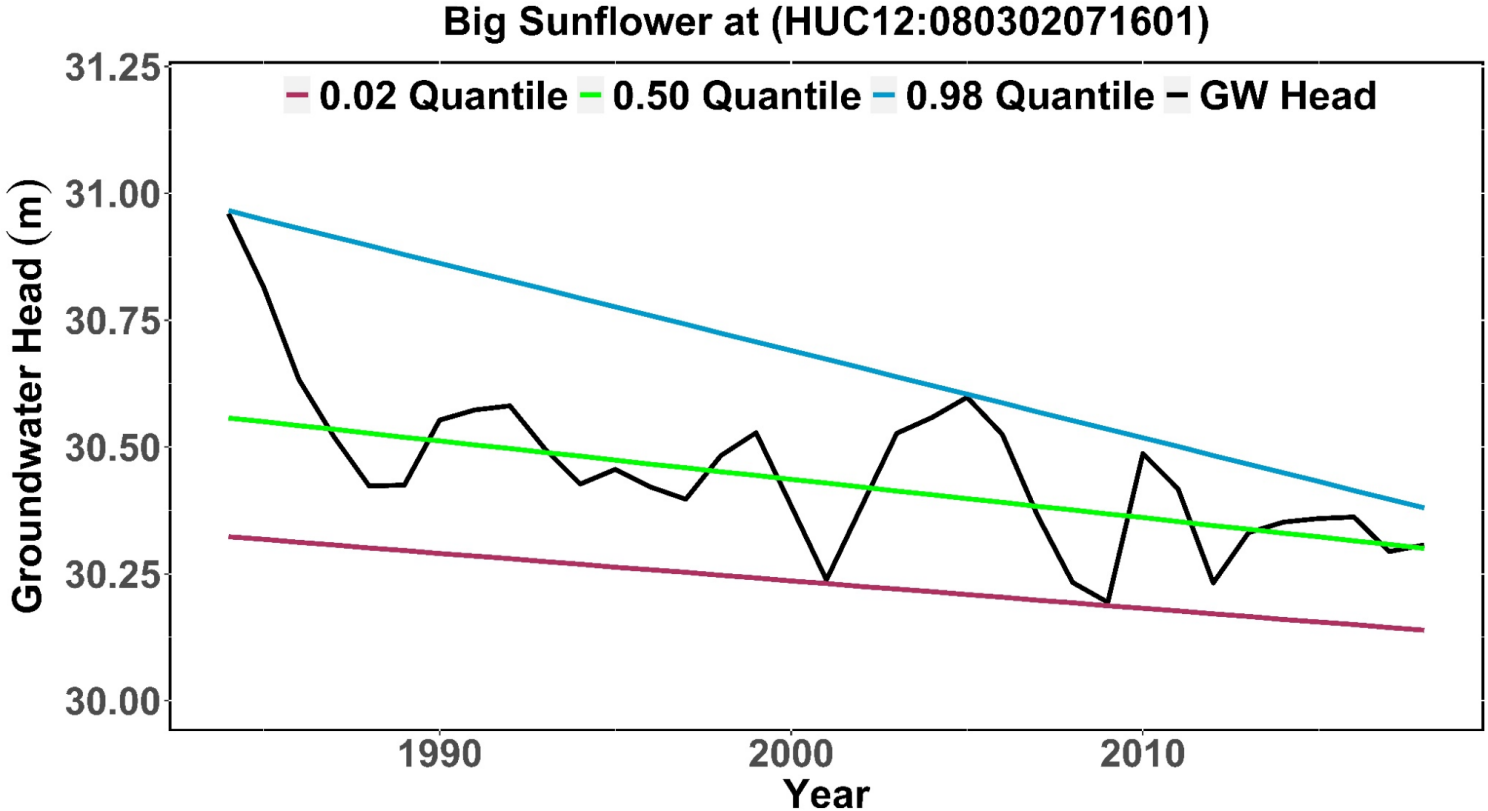
Annual simulated groundwater head with quantile trends of extremely dry (0.02 quantile), average condition (0.50 quantile), and extremely wet (0.98 quantile) for Big Sunflower River at (HUC12:080302071601: subbasin).

The time dependency (i.e., trend) of the specified quantile value is indicated by the two linear coefficients. Figure 5 illustrates various quantile trend lines of simulated groundwater head for (HUC12:080302071601) within Big Sunflower River, revealing a distinct dispersion. Quantiles offer insight into particular segments of data distribution. The 0.02 quantile signifies extremely dry conditions, as it corresponds to the lower tail of the data distribution where groundwater levels are at their lowest. The 0.98 quantile depicts extremely wet conditions, exhibiting the upper tail of the distribution where groundwater levels are at their highest. The 0.50 quantile, or median, correspond to average conditions, presenting a measure of the central tendency of groundwater levels. It is important to acknowledge that while the preceding example seeks to illustrate how QR expands upon the concept of linear regression, the QR approach is not necessarily linear or parametric.

In this study, we select its parametric, linear version to facilitate the measurement of trends. Bootstrap approaches have been developed to assess the significance of the fit in quantile regression. We use the R package *‘quantreg’*^88^, which incorporates both fitting methods and significance testing methodologies. The null hypothesis for this analysis asserts that no trend exists in the specified quantile over time (i.e., the slope of the trend line is zero), whereas the alternative hypothesis suggests the presence of a significant trend.

In this investigation, we used the calibrated SWAT + models to investigate long-term trends of groundwater head, groundwater evapotranspiration, groundwater pumping, groundwater recharge, and groundwater seepage for extremely wet (0.98 quantile trend), extremely dry (0.02 quantile trend), and average conditions (0.50 quantile trend). The predictors in the analysis are time, revealing the independent variable, whereas the predictands are groundwater head, groundwater pumping, groundwater evapotranspiration, groundwater recharge, and groundwater seepage. We employed a two-sided hypothesis test at a level of significance of 5% (*p* < 0.05) to evaluate the statistical significance of trends. P-values for each trend were computed with bootstrap methods incorporated in the R package *‘quantreg’*. Trends with p-values below 0.05 were deemed statistically significant and are emphasized in the results.

## Results and discussion

### System responses: streamflow, groundwater head, and surface ET

**Fig. 6:**
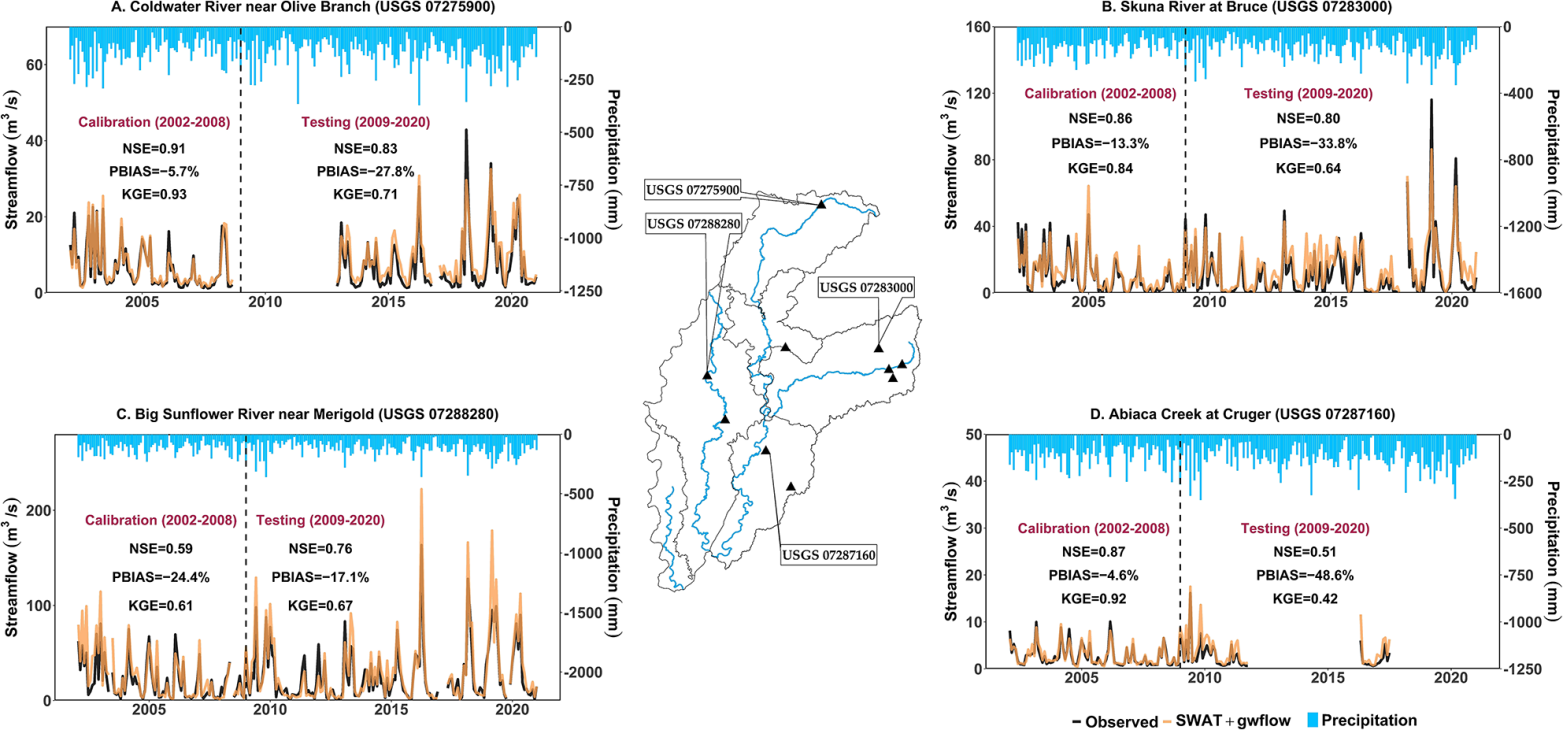
Monthly simulated streamflow for SWAT + model for four chosen river gage stations within the MAP watershed. Statistical model performances (NSE, KGE and PBIAS) are presented for each gage location.

Table 4 shows the statistical performance (i.e., NSE, R^2^, PBIAS, and KGE) of monthly simulated streamflow at 10 river gage locations for the calibration (2002–2008) and testing (2009–2020) periods within the study area. The streamflow results show acceptable to good performance at most locations, with NSE values ranging from 0.49 to 0.91 for the calibration period and 0.44 to 0.92 for the testing period. Figure 6 reveals the monthly simulated streamflow at four chosen locations within different sub-watersheds. By utilizing a desktop computer, an Intel^®^ Core™ i7-10700 CPU @ 2.90 GHz with 64 GB RAM, the computational runtime for running the SWAT + model during the 9-year calibration period (2000–2008) differed throughout the watersheds, indicating variations in model complexity and scale. The estimated simulation timings for the watersheds were as follows: Tallahatchie (6 min), Coldwater (15 min), Yalobusha (8 min), Upper Yazoo (7 min), Big Sunflower (56 min), and Deer-Steele (7 min).

**Fig. 7:**
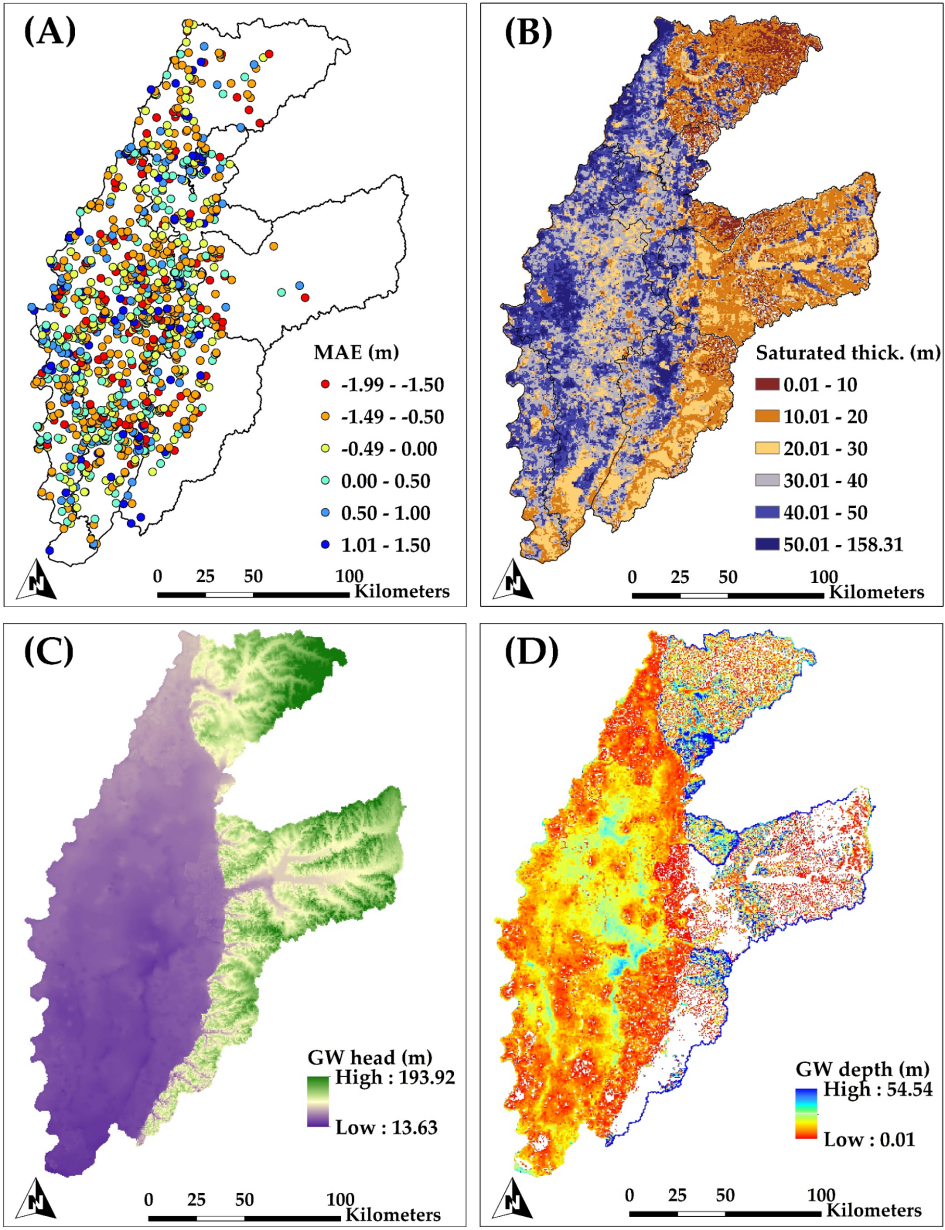
Maps demonstrate (a) the statistical model’s performance the mean absolute error (MAE) in meters (m), for groundwater level throughout the simulation period from 2000 to 2020; (b) saturated thickness (m) for year 2020; (c) groundwater head (m) for year 2020; and (d) groundwater depth (m) for year 2020.

Figure 7a depicts the statistical matrix, indicated by the Mean Absolute Error (MAE), of the annual groundwater levels at 1155 wells within the study region from 2000 to 2020. The MAE results demonstrate a commendable level of accuracy, exhibiting a residual error of less than 1.5 m in the comparison of simulated and measured average annual groundwater head at each USGS monitoring well site. Nevertheless, certain specific places demonstrate a marginally greater error, approximately 2.0 m. It is essential to acknowledge that these disparities are comparatively insignificant in relation to the total thickness of the saturated aquifer. Figure 7b illustrates a map of saturated thickness, defined as the vertical distance between the bedrock and the water table, for the study watersheds in the final year of simulation (2020). The regional distribution of saturated thickness mirrors the thickness of the unconfined aquifer depicted in Fig. 2D. Nevertheless, fluctuations in groundwater head and depth (Fig. 7c and d) within each watershed explain the disparities in saturated thickness. The saturated thickness metric offers insight into available groundwater storage, with greater thickness signifying increased storage capacity, an essential consideration for evaluating long-term aquifer health.

**Fig. 8:**
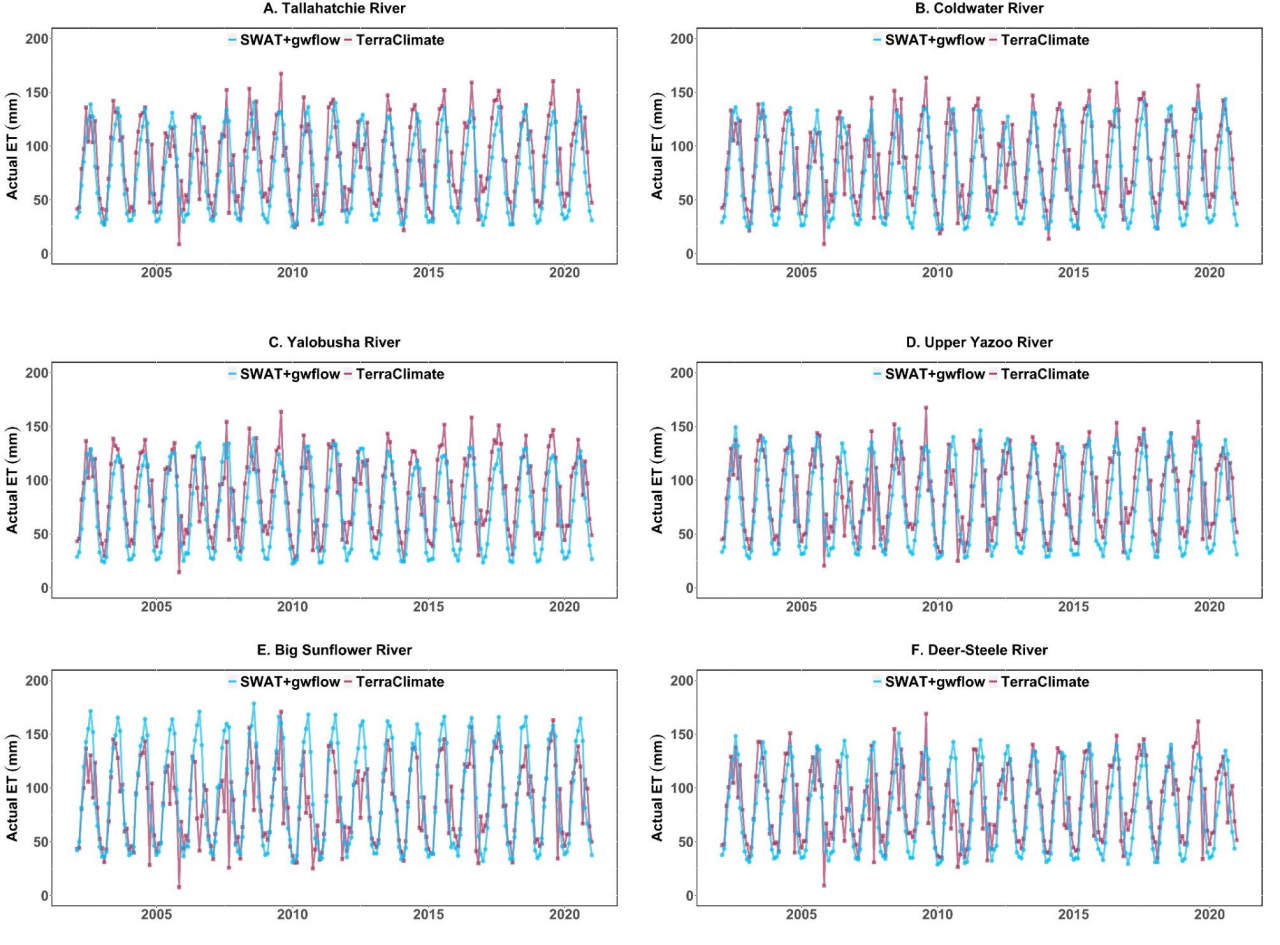
Watershed average monthly comparison of simulated actual ET (SWAT+) with TerraClimate data for (2002–2020) for the six HUC8 watersheds.

Figure 8 exhibits a comparison of monthly actual evapotranspiration (ET) between the SWAT + models and TerraClimate data across six sub-basins in the study region for period of 2002–2020. Each watershed is assessed discretely to evaluate the model’s accuracy in replicating seasonal and interannual ET patterns. The simulated SWAT + models capture the seasonal cycles of ET, with peaks in warmer months and lows in cooler months, suggesting that the model generally aligns with observed seasonal timing. However, there are consistent differences in ET magnitude with TerraClimate often demonstrating higher peak values than SWAT+, especially during summer.

The simulated SWAT models is consistent with TerraClimate for the timing and peaks simulations, indicating that the calibrated models are successful and may serve as a reference for other regions. As opposed to, Big Sunflower exhibits certain differences possibly attributable to localized irrigation techniques or crop-specific requirements that may not be entirely represented in the model. While SWAT + models effectively illustrate broad ET patterns, calibrating ET parameters within sub-basins could improve model precision, especially during peak demand periods, thereby offering a more accurate depiction of water utilization in these heavily irrigated areas.

### Hydrologic fluxes in the MAP

Table 5 reveals the average annual hydrologic fluxes for six HUC8 watersheds within MAP. The surface runoff and surface evapotranspiration are dominant fluxes signifying the combined influence of natural vegetation requirements, significant volumes of water traversing the surface, and probable evaporation resulting from the humid climate. The consistent trends seen across the watersheds indicate that evapotranspiration and surface runoff are crucial water channels, impacting the volume of water that infiltrates into groundwater systems and, in turn, influencing groundwater storage levels.

Groundwater recharge, seepage, and irrigation-related fluxes exhibit substantial variability among watersheds, mostly driven by differing irrigation demands and groundwater utilization. Groundwater recharge is significantly high in the Yalobusha watershed (333.7 mm), but it is considerably diminished in Deer-Steele (43.5 mm), indicating that certain watersheds experience greater recharge due to particular hydrogeological conditions or reduced groundwater extraction intensity. Groundwater seepage is higher in Yalobusha (217.0 mm), suggesting that natural infiltration enhances groundwater levels in this region, in contrast to the reduced seepage observed in locations such as Big Sunflower and Deer-Steele. The pumping and surface water irrigation rates reveal disparities, with watersheds such as Tallahatchie and Upper Yazoo exhibiting significant pumping (13.7 and 13.9 mm) and surface water irrigation (23.5 and 23.0 mm), emphasizing their dependence on groundwater for agricultural requirements in this heavily irrigated area. The disparities are essential for assessing groundwater storage depletion, since regions with significant extraction but minimal recharge, such as Deer-Steele, may be more susceptible to exhaustion. Conversely, watersheds with elevated recharge, like Yalobusha, may maintain a more sustainable equilibrium between extraction and replenishment, hence diminishing the risk of prolonged groundwater storage depletion.

**Fig. 9:**
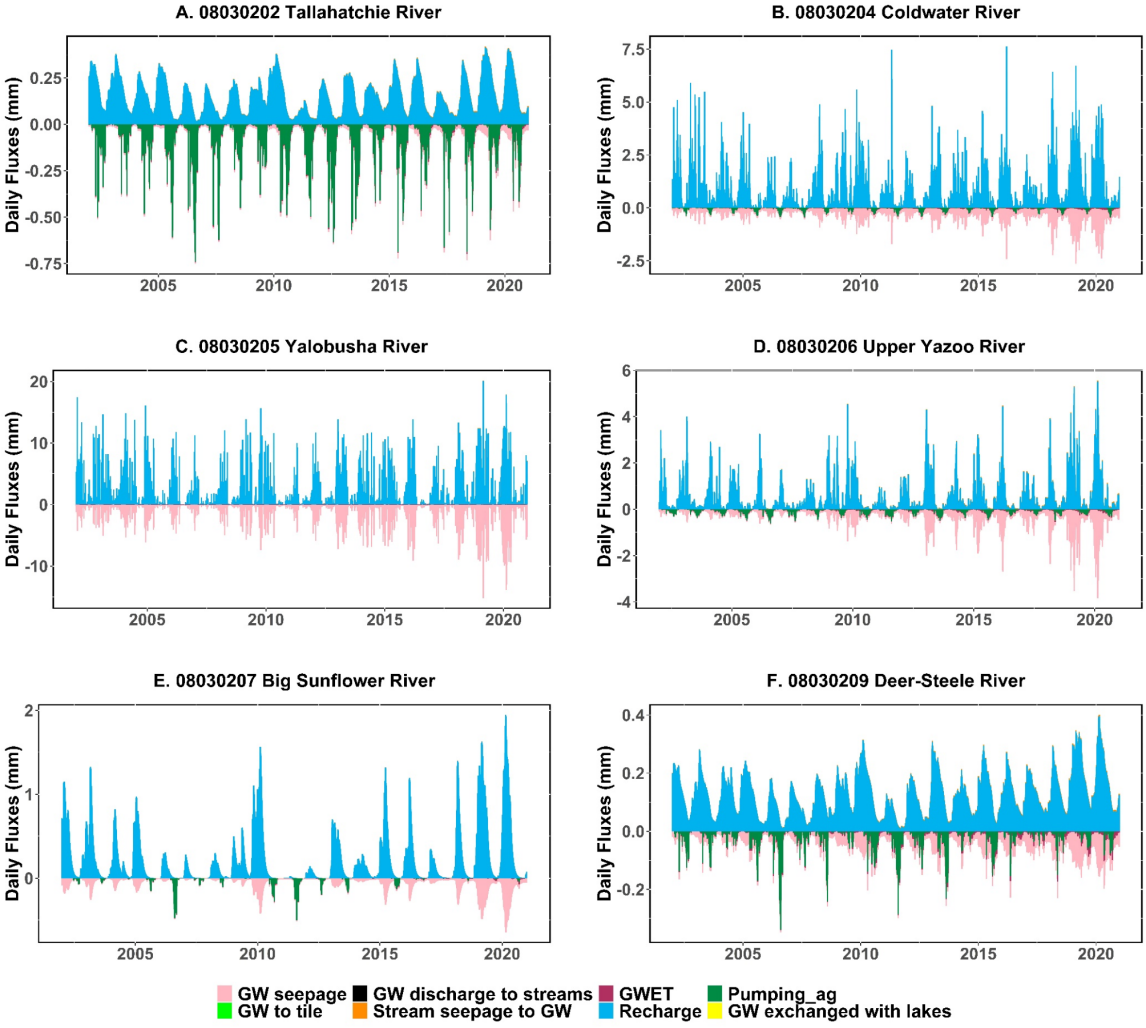
Daily average groundwater fluxes for 6 HUC8 of MAP for the simulation period from 2002 to 2020.

Figure 9 shows the daily average groundwater fluxes for six HUC8 watersheds in the Mississippi Alluvial Plain, offering a comprehensive overview of diverse groundwater interactions. Principal fluxes encompass groundwater seepage, groundwater discharge to streams, groundwater evapotranspiration (GWET), recharge, agricultural pumping, and stream seepage to groundwater. The panel for each watershed illustrates the variability and seasonality of groundwater fluxes, providing context for the yearly average fluxes presented in Table 5.

Considering that the majority of pumping activities occur inside the Big Sunflower watershed, as evidenced by field observations and other data sources, Fig. 9 may elucidate the reasons for this phenomenon. The substantial agricultural demand and extensive irrigation requirements in Big Sunflower may necessitate this heightened pumping. Figure 9E illustrates persistent agricultural pumping activity year-round, corresponding with the watershed’s extensive agricultural operations. The consistent pumping pattern in Big Sunflower indicates a significant dependence on groundwater resources for irrigation, likely necessitated by the soil and crop kinds that demand continuous water supply. Moreover, the diminished availability of surface water resources relative to other watersheds may potentially lead to an increased dependence on groundwater in Big Sunflower.

### Trend analysis results

**Fig. 10:**
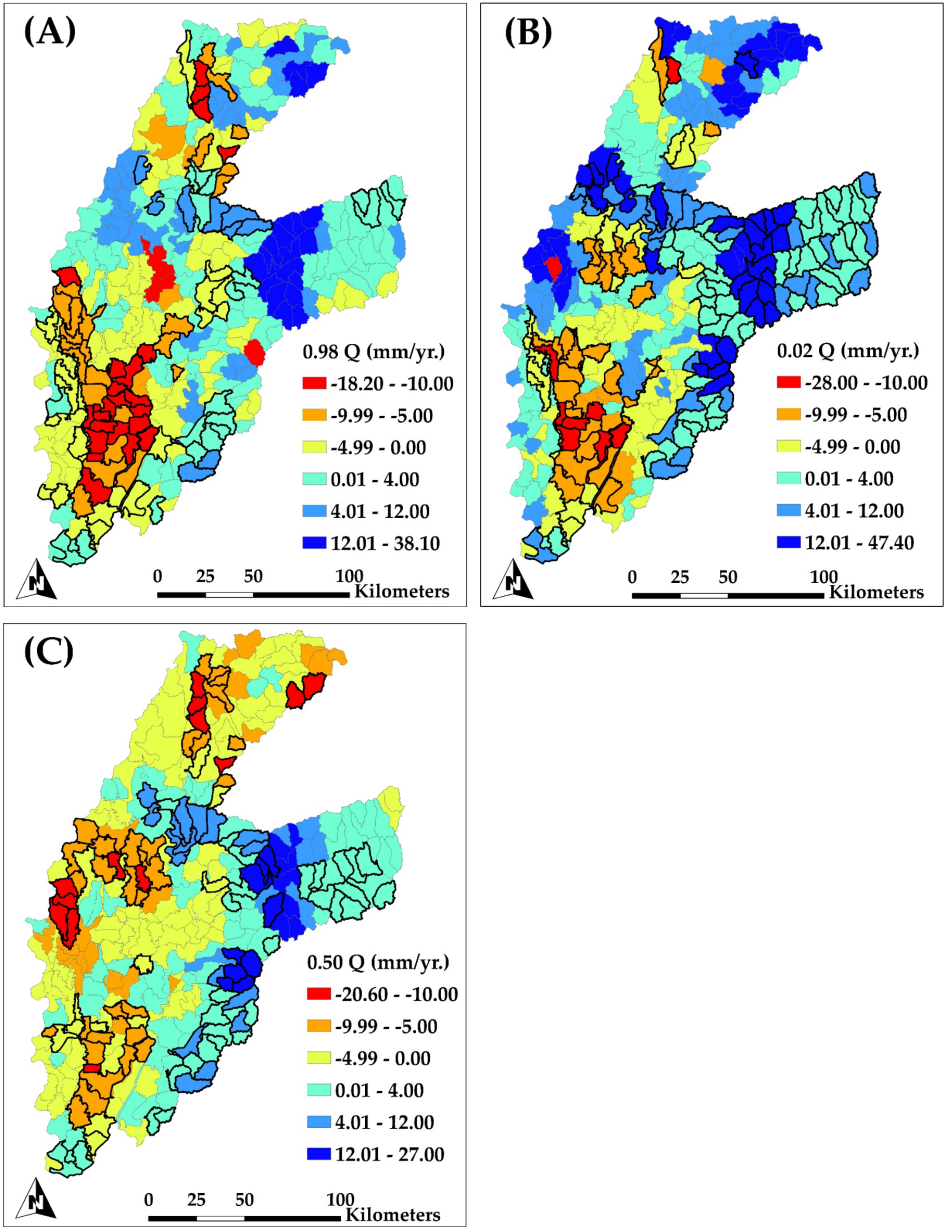
Spatial distribution of the trend of simulated groundwater head for each subbasin (HUC12 level) for the period of 1984–2018 for (A) upper extremes trends (0.98 Quantile); (B) lower extremes trends (0.02 Quantile); and (C) median trends (0.50 Quantile). Highlighted subbasins indicate a significant trend.

Figure 10 illustrates the spatial distribution of annual groundwater head trends within the examined region for each subbasin (HUC12), evaluated via quantile regression at three quantiles: 0.98 (Fig. 10A), 0.02 (Fig. 10B), and 0.5 (Fig. 10C). Each quantile elucidates distinct facets of groundwater head variability, providing a thorough perspective on fluctuations in groundwater levels. Figure 10A, illustrating the 0.98 quantile trend (highlighting extreme wet years), reveals significant reductions in groundwater head throughout multiple HUC12 subbasins in the alluvial plain on the western side of the study area. The reductions are significant, with a declining trend of − 18.20 mm/yr. The most significant losses occur in the alluvial plain in the western region, where intense groundwater pumping for irrigation has led to considerable depletion of groundwater levels. Although a few regions within this quantile exhibit statistically insignificant increases (38.10 mm/yr in the mountainous region on the eastern part), the prevailing trend in the alluvial plain is a fall, emphasizing the effects of pumping on groundwater resources.

Figure 10B emphasizes the 0.02 quantile trend (highlighting extreme dry years), which reflects the most pronounced reductions in groundwater head. The western of the study region, especially the boundary subbasins in the alluvial plain, exhibits significant reductions, with values decreasing by as much as − 28.00 mm/yr. These reductions indicate regions where groundwater resources are extensively utilized, presumably due to agricultural irrigation demands. On the contrary, the mountainous regions on the eastern exhibit modest increases in groundwater head, attaining up to 47.40 mm/yr., presumably attributable to natural recharge and less pumping.

Figure 10C demonstrates the median trends (0.50 quantile), offers a comprehensive perspective on groundwater head fluctuations throughout the study area. The reductions are again significant in the alluvial plain subbasins, with rates reaching as high as − 20.60 mm/yr. in certain boundary regions. In contrast to the generally declining tendency in the alluvial plain, certain mountainous regions are showing small rises (up to 27.00 mm/yr.). This trend indicates that groundwater resources in the alluvial plain are experiencing considerable stress due to extensive pumping.

**Fig. 11:**
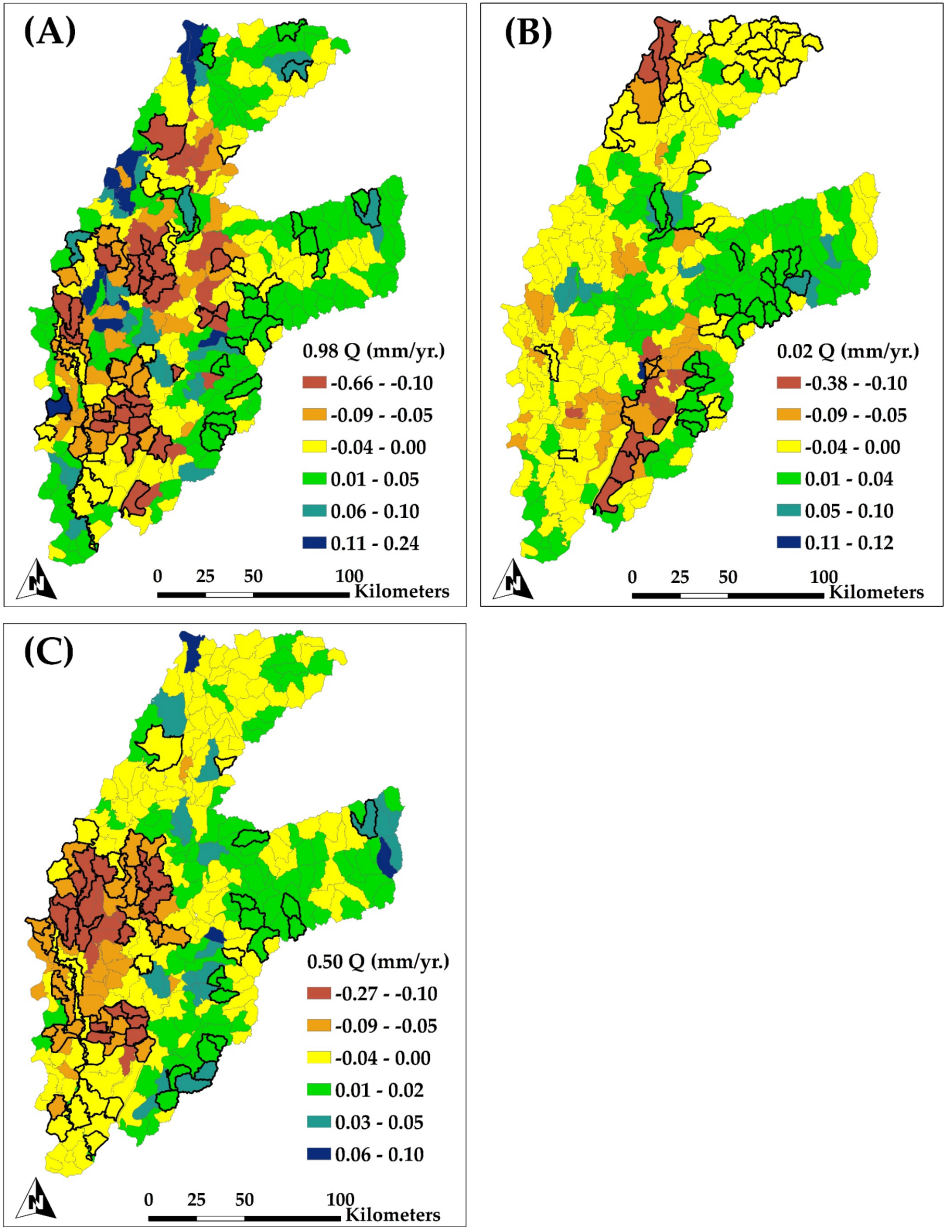
Spatial distribution of the trend of simulated groundwater evapotranspiration for each subbasin (HUC12 level) for the period of 1984–2018 for (A) upper extremes trends (0.98 Quantile); (B) lower extremes trends (0.02 Quantile); and (C) median trends (0.50 Quantile). Highlighted subbasins indicate a significant trend.

Figure 11 shows the spatial distribution of annual trends in simulated groundwater evapotranspiration (GW ET) throughout HUC12 subbasins. The quantile trends offer insights into the variability of groundwater evapotranspiration trends, emphasizing regions where groundwater is increasingly depleted through evapotranspiration, a feature that may exacerbate total groundwater storage depletion, particularly when associated with decreasing groundwater head trends.

Figure 11A (0.98 quantile) shows significant decrease trend in groundwater evapotranspiration across HUC12 subbasins predominantly located in the alluvial plain regions on the eastern of study region. Certain subbasins exhibit reductions as extreme as − 0.66 mm/yr. This pattern indicates that groundwater is less accessible for evapotranspiration in these regions, presumably due to continuous groundwater depletion. The reductions in groundwater evapotranspiration correspond with the notable falls in groundwater head in the same regions, suggesting that as groundwater storage decreases, the potential for groundwater-supplied evapotranspiration also diminishes, a tendency that intensifies overall store depletion. In contrast, slight increase in groundwater evapotranspiration are noted in the mountainous subbasins on the east, with rises reaching 0.24 mm/yr. This indicates a more stable groundwater system in these regions, with sufficient accessible groundwater to sustain evapotranspiration.

Figure 11B illustrates the lower extreme trends (0.02 quantile) in groundwater evapotranspiration, indicating the regions where reductions in groundwater-supplied evapotranspiration are most significant. In the alluvial plains, several subbasins demonstrate declines of up to − 0.38 mm/yr. in groundwater evapotranspiration. This significant decline aligns with the drastic reductions in groundwater head found in the same area, underscoring the relationship between lowered groundwater levels and decreased evapotranspiration. In regions where groundwater levels are already compromised by excessive pumping, the reduction in groundwater evapotranspiration underlines the diminishing availability of groundwater to support evapotranspiration processes, hence intensifying storage depletion.

Figure 11C presents the median trends (0.50 quantile) in groundwater evapotranspiration over the study area, representing more conventional settings. At the median level, several subbasins in the alluvial plain exhibit reductions in groundwater evapotranspiration, with values declining to − 0.27 mm/yr. This reduction indicates that groundwater depletion is persistently impairing the capacity of groundwater to sustain evapotranspiration in these areas. The correlation between declined groundwater evapotranspiration and decreasing groundwater head trends indicates a feedback loop: as groundwater levels decline, the availability of water for evapotranspiration decreases, potentially diminishing groundwater recharge capacity and exacerbating groundwater storage depletion. Conversely, the mountainous areas on the east exhibit marginal increases in groundwater evapotranspiration (up to 0.10 mm/yr.), signifying steady groundwater supplies.

**Fig. 12:**
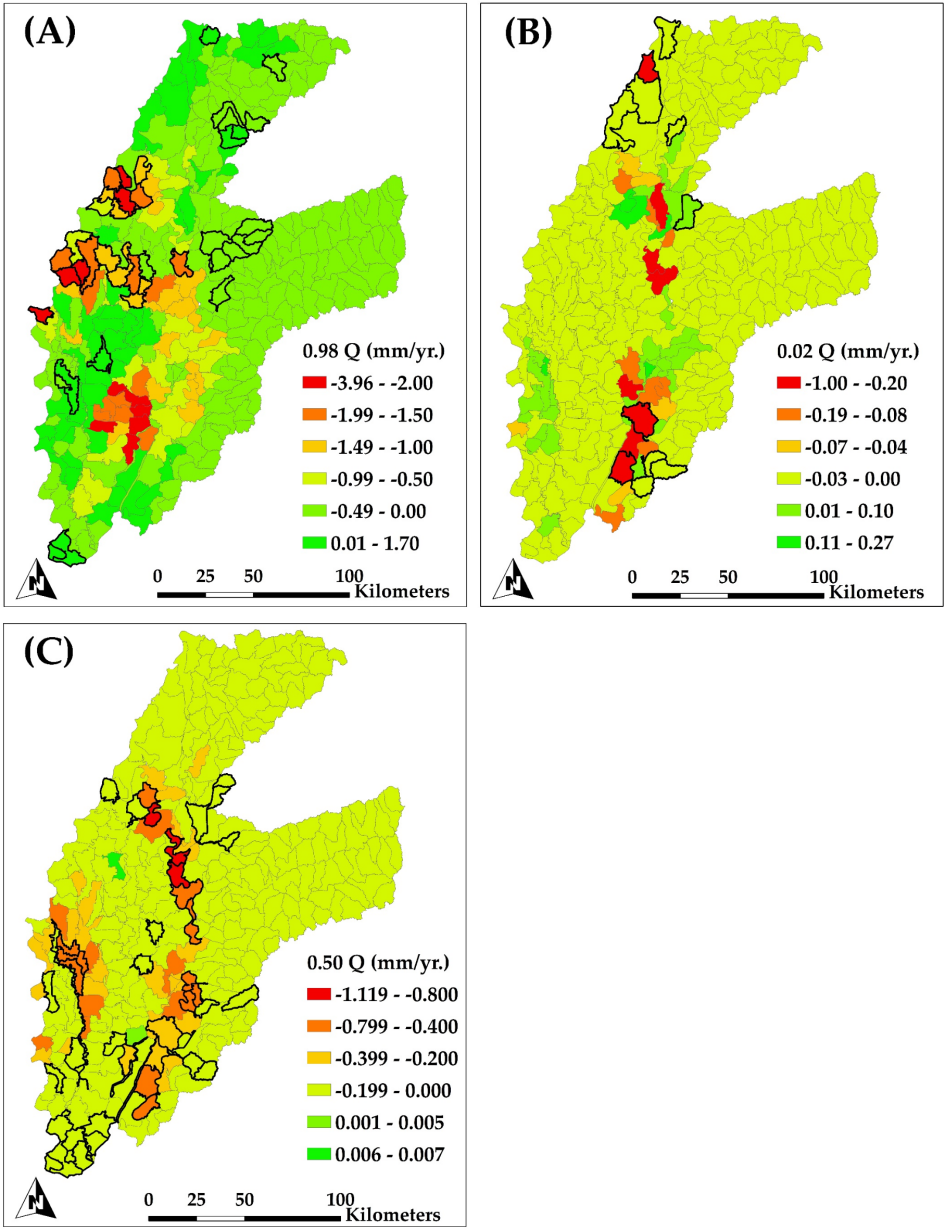
Spatial distribution of the trend of simulated groundwater pumping for each subbasin (HUC12 level) for the period of 1984–2018 for (A) upper extremes trends (0.98 Quantile); (B) lower extremes trends (0.02 Quantile); and (C) median trends (0.50 Quantile). Highlighted subbasins indicate a significant trend.

Figure 12 depicts the spatial distribution of annual trends in simulated groundwater pumping throughout HUC12 subbasins, examined by quantile regression at the 0.98 quantile (Fig. 12A), 0.02 quantile (Fig. 12B), and 0.50 quantile (Fig. 12C). This figure emphasizes areas where groundwater pumping trends show significant variability, specifically in the context of groundwater. Figure 12A (0.98 quantile) illustrates regions with significant reductions in groundwater pumping. The regions shown in red and orange exhibit reductions in pumping rates of up to − 3.96 mm/yr. The diminishing pumping trend in these subbasins, predominantly located in the alluvial plain on the western of the study region, may be attributable to decreased groundwater availability or regulatory constraints resulting from storage depletion. The reduction in pumping corresponds with the documented declines in groundwater head, as illustrated in the preceding groundwater head trend maps (Fig. 10). As groundwater levels diminish, the decreased supply of groundwater may restrict pumping capacity, exacerbating these declining trends in pumping. Concurrently, several regions demonstrate slight increases in pumping (up to 1.70 mm/yr.), which are predominantly dispersed and may indicate localized demands or particular agricultural practices.

Figure 12B (0.02 quantile) depicts regions where groundwater extraction rates have shown minor yet consistent reductions. Significant declines in pumping, reaching − 1.00 mm/yr., are again apparent in the alluvial plain, especially within boundary subbasins. The constant reductions in pumping align with regions of elevated irrigation demand that are probably encountering difficulties in sustaining groundwater supply due to persistent extraction pressures. The convergence of diminished groundwater levels, decreased groundwater evapotranspiration, and reduced extraction in these places highlights the collective impact of groundwater depletion in heavily irrigated regions.

Figure 12C displays the median trends in groundwater pumping, reflecting standard conditions throughout the study region. Pumping is somewhat declining in several subbasins on the alluvial plain, with values reaching − 1.12 mm/yr. This pattern highlights the feedback loop created by groundwater depletion and is consistent with largely notable declines in groundwater levels and evapotranspiration observed in these regions. As groundwater diminishes, pumping capacity is inherently limited. Conversely, in some subbasins, there are minor positive trends in pumping (up to 0.007 mm/yr.), indicating regions where groundwater pumping remains viable. The possible reasons for the noted decline in groundwater pumping include alterations in precipitation patterns, changes in agricultural water demands, and significant depletion of groundwater levels. These factors restricted the ability of pumping, indicating physical constraints in the water supply rather than a decrease in demand.

**Fig. 13:**
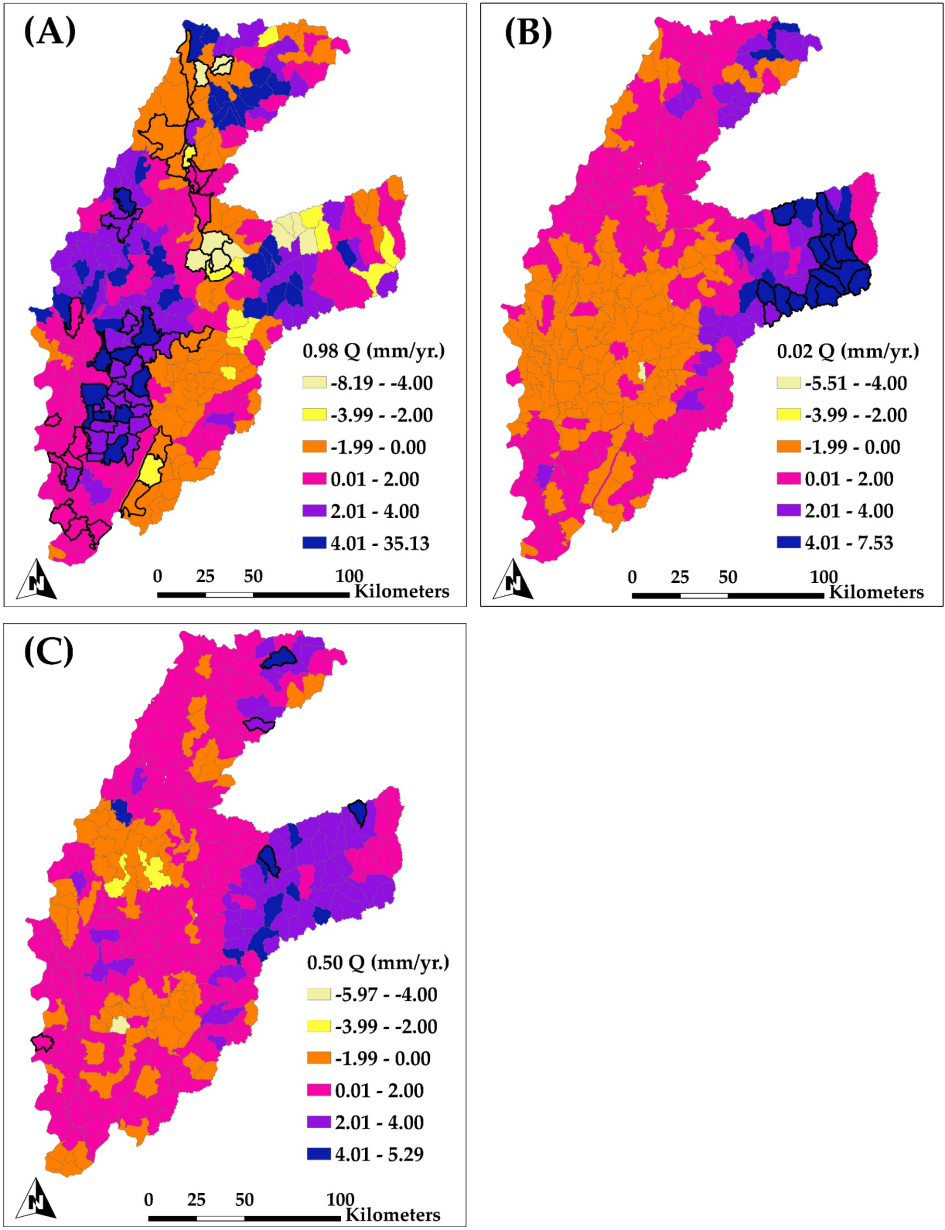
Spatial distribution of the trend of simulated groundwater recharge for each subbasin (HUC12 level) for the period of 1984–2018 for (A) upper extremes trends (0.98 Quantile); (B) lower extremes trends (0.02 Quantile); and (C) median trends (0.50 Quantile). Highlighted subbasins indicate a significant trend.

Figure 13A (0.98 quantile) illustrates regions exhibiting both positive and negative trends in groundwater recharge. The alluvial plain exhibits significant increases in recharge rates, attaining up to 35.13 mm/yr. This indicates specific regions where recharge has increased, either due to irrigation return flow or precipitation events enhancing infiltration. Nonetheless, certain subbasins are showing reductions in recharge, as low as − 8.19 mm/yr., which may be attributed to a slight reduction in precipitation, particularly in the eastern region. An in-depth analysis of particular subbasins (e.g., HUC12) indicates minor decreases in groundwater head trends, which correspond with the noted recharge trends, so reinforcing the model’s accurate depiction of hydrological processes. Regions with high recharge may provide some mitigation against groundwater depletion; however, the significant reductions in groundwater levels demonstrate that this recharge is inadequate to counteract the persistent loss of storage. Interestingly, these opposing trends between recharge and groundwater head indicate localized recharge enhancements that may not significantly raise groundwater levels due to continued high pumping demands or insufficient vertical connectivity between recharge zones and deeper aquifers.

Figure 13B (0.02 quantile) shows locations characterized by predominantly declined recharge patterns, where the majority exhibit minimal positive alterations in recharging and several areas demonstrate negative trends, with value reaching − 5.51 mm/yr. in several subbasins. The diminished recharge in some subbasins of the alluvial plain indicates a restricted ability for natural replenishment, potentially resulting from the depletion of groundwater levels or inadequate infiltration beneath irrigated farms. In this quantile, the observed negative recharge trends align with the significant drops in groundwater levels, highlighting regions where the interplay of reduced infiltration and excessive groundwater pumping exacerbates storage losses. The persistent low recharge coupled with decreasing groundwater head trends underscores that recharge alone is insufficient to offset storage losses in regions significantly impacted by groundwater extraction.

Figure 13C (median quantile) highlights standard patterns in groundwater recharge, with moderate increases in certain subbasins (up to 5.29 mm/yr. in blue regions) while also revealing reductions in other locations, with values as low as − 5.97 mm/yr. in yellow regions. Recharge patterns in the alluvial plain are not uniform; some subbasins do exhibit a slight upward trend in recharge, but this is likely not enough to offset the high rates of groundwater pumping. The median trend indicates that although recharging occurs, its extent and spatial distribution do not entirely counteract the reductions in groundwater storage noted in the alluvial plain. The remarked reduction in groundwater recharge may stem from multiple interconnected sources. A prospective decline in precipitation may restrict surface water availability for infiltration, thereby diminishing recharge rates. Moreover, ongoing groundwater pumping has probably diminished water tables, decreased hydraulic gradients and consequently restricted the natural flow of water from the soil into deeper aquifers. Notably, the spatial inconsistency in recharge trends underscores the complexity of recharge mechanisms, particularly in regions heavily influenced by irrigation return flows, altered soil properties, and local precipitation variability. Soil conditions in these regions may have been modified by extensive irrigation, resulting in compaction or diminished soil permeability in certain fields, which might restrict water infiltration. The complex web of factors affecting recharge rates is further illustrated by the interaction between precipitation variability, irrigation practices, and changes in soil conditions. This confirms the challenges of maintaining groundwater levels in the face of current pumping demands.

**Fig. 14:**
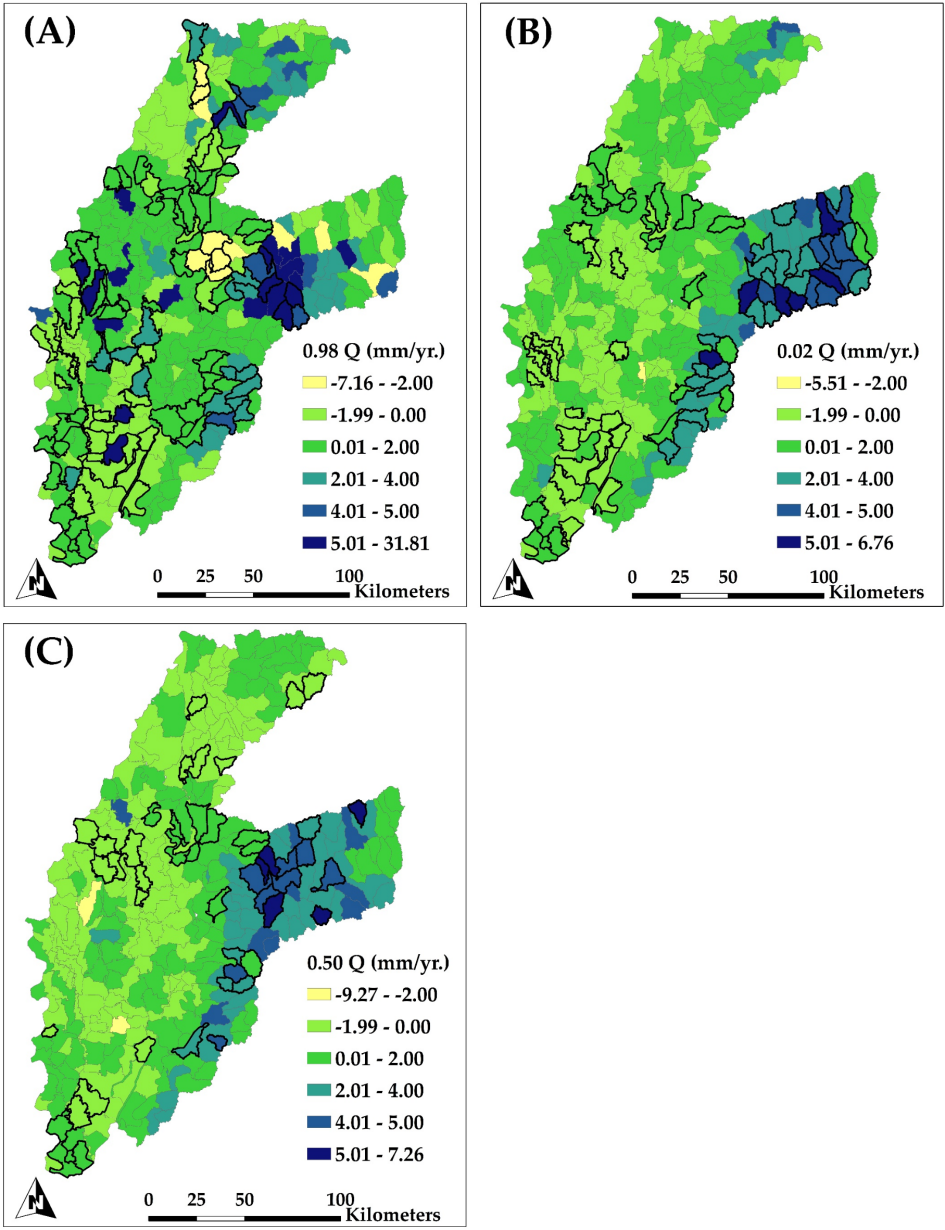
Spatial distribution of the trend of simulated groundwater seepage for each subbasin (HUC12 level) for the period of 1984–2018 for (A) upper extremes trends (0.98 Quantile); (B) lower extremes trends (0.02 Quantile); and (C) median trends (0.50 Quantile). Highlighted subbasins indicate a significant trend.

Figure 14A (0.98 quantile) reveals areas exhibiting significant increase in groundwater seepage, especially on the eastern region of the map (mountainous regions), where seepage rates attain a maximum of 31.81 mm/yr. The rise in seepage may be ascribed to high recharge rates in mountainous areas, where natural replenishment augments subsurface flows. In contrast, certain subbasins within the alluvial plain exhibit decreasing seepage tendencies, with values as low as − 7.16 mm/yr. The reductions in seepage correspond with regions exhibiting substantial dips in groundwater head, suggesting that lowered water tables restrict seepage, hence exacerbating groundwater storage depletion. In some areas, decreased seepage may indicate the groundwater system’s reduced capacity to sustain outflows, intensifying store depletion.

Figure 14B (0.02 quantile) shows areas with insignificant seepage trends, concentrating on regions with constant or negligible alterations in seepage rates. In this lower quantile, the map indicates significant increases in seepage, with values reaching 6.76 mm/yr. In the alluvial plain, decreases in groundwater seepage are apparent, indicating that the groundwater table has fallen below the channel stage, thereby lessening the likelihood of groundwater discharge to streams. The ongoing reduction in seepage emphasizes the effects of declining groundwater levels, as streams are unable to receive discharge from the groundwater system, hence intensifying the pressure on groundwater reserves.

Figure 14C presents median quantile of seepage trends, illustrating standard conditions throughout the study region. In the alluvial plain, seepage patterns exhibit variability, with minor rises in certain regions (up to 7.26 mm/yr.) and declines in others, reaching − 9.27 mm/yr. The minor reductions in seepage within the alluvial plain underscore the feedback mechanism between decreasing groundwater levels and decreased seepage, which can affect groundwater head and storage capacity. In contrast, the eastern mountainous regions show seepage patterns that are more consistent or somewhat positive, which supports the observation of stable groundwater levels in these areas. Table S1 in Supporting Information file presents a summary of statistically significant quantile trends for different hydrological variables across each of the six watersheds in MAP.

The results of this study on groundwater storage depletion in the Mississippi Alluvial Plain (MAP) align with other research emphasizing the effects of agricultural extraction and irrigation requirements on groundwater reduction in the area. The Mississippi Alluvial Plain (MAP) has been undergoing substantial groundwater storage depletion, chiefly attributable to agricultural removal and irrigation requirements. Leaf et al. (2023)^39^ simulated groundwater flow in the Mississippi Delta, estimating daily groundwater storage losses of around 10,000 m³ during peak years such as 2005 and 2006. This corresponds with identical findings by Sumner and Wasson (1990)^89^, wherein models indicated a net storage depletion of 360 million gallons per day from April 1981 to April 1983 in the northwestern Mississippi aquifer basin. These findings indicate a sustained depletion trend in MAP, exacerbated by low groundwater recharge rates and climate variability.

This issue has been illuminated by remote sensing and satellite-based approaches like GRACE (Gravity Recovery and Climate Experiment). By comparing GRACE-derived data on fluctuations in groundwater storage with in-situ measurements, Rateb et al. (2020)^6^ found that groundwater storage decreases significantly each year, which is in line with the conclusions from simulations. The results show that the MAP region experiences annual storage deficits regardless of the recharge rates of groundwater, which are exacerbated by irrigation practices that do not conserve groundwater. Due to the continued decline in aquifer levels caused by conventional methods, Yang et al. (2019)^90^ anticipated that innovative management strategies will be required to meet the irrigation demands of the 21st century.

To alleviate additional storage depletion, some studies have proposed tactical modifications to water management. Rashid (2014)^91^ and Nelson et al. (2022)^92^ investigated the effects of modifying irrigation practices, observing that conservation-oriented methods could markedly diminish groundwater depletion. Rashid’s model examined ideal withdrawal thresholds to preserve the aquifer, whereas Nelson’s research on the capabilities of big data analytics highlighted the advantages of precision irrigation. Accordingly, groundwater supplies in the MAP will continue to face unsustainable depletion unless there are major management actions.

This research provides a unique contribution to the comprehension of groundwater storage depletion in the Mississippi Alluvial Plain (MAP) by the application of surface-subsurface hydrologic modeling methods and quantile regression analysis. This research offers a comprehensive, quantile-based analysis of groundwater head trends, evapotranspiration, recharge, and seepage, distinguishing itself from prior studies that predominantly utilized conventional methods or concentrated on general groundwater trends, by capturing both extreme and median responses across the MAP. By incorporating fine-scale spatial and temporal variability, our study reveals unique trends at the subbasin level that have been overlooked in larger-scale regional assessments. Furthermore, this analysis differs from others that have only focused on groundwater extraction since it incorporates many hydrologic fluxes to provide a comprehensive evaluation of the factors that contribute to storage depletion.

This study’s findings underscore the urgent necessity for comprehensive groundwater management methods in the Mississippi Alluvial Plain (MAP). Effective management should incorporate a comprehensive strategy that integrates innovative irrigation technologies, including drip and smart irrigation systems, to improve water use efficiency and minimize groundwater extraction. Employing surface water during wet periods can reduce dependence on groundwater, while recharge techniques, such as rainwater harvesting, can alleviate diminishing store levels. Implementing enduring monitoring systems, underpinned by hydrological models such as SWAT, would facilitate data-informed decision-making and adaptive management. Furthermore, policy and economic incentives, including subsidies for efficient irrigation methods or tiered water pricing, may encourage stakeholders to embrace sustainable practices. Collectively, these solutions establish a comprehensive framework for mitigating groundwater depletion in the MAP while maintaining agricultural output and resource sustainability.

## Study limitations

This study offers significant insights into groundwater dynamics in the Mississippi Alluvial Plain utilizing the SWAT + model with the enhanced *gwflow* module. Nonetheless, some limitations must be recognized, which offer significant background for evaluating the results and avenues for further research:

### Spatial resolution restrictions

The model utilizes a 500 m grid resolution for computational efficiency; nevertheless, it may not adequately describe finer-scale phenomena, like localized groundwater-surface water interactions or field-scale variability near rivers. Future research may benefit from utilizing the unstructured version of the *gwflow* module to increase spatial representation and boost the precision of localized hydrological processes.

### Simplified groundwater representation

The groundwater system is depicted as a single-layer, vertically integrated aquifer, disregarding vertical heterogeneity and deeper groundwater dynamics. This simplification emphasizes shallow groundwater dynamics essential for irrigation, but it limits the assessment of deeper aquifer responses and extended lateral groundwater flow throughout the basin.

### Temporal scale of calibration data

The model is tested with annual groundwater head data, which may obscure short-term fluctuations. Subsequent research should investigate the application of higher-frequency (e.g., monthly) groundwater head data for calibration and testing purposes.

### Recharge from non-field HRUs

Recharge from cultivated fields to the unconfined aquifer is precisely modeled; however, recharge from non-field hydrologic response units is not spatially explicit. The absence of delineation of these HRUs in the NAM leads to the calculation of recharge for non-field areas based on the average recharge rate of the 12-digit catchment (i.e., HUC12).

### Boundary condition simplifications

Groundwater fluxes at the watershed boundary are modeled utilizing a boundary condition technique. The groundwater head in boundary cells is presumed to remain constant at its starting value at the commencement of the simulation. Fluxes are modeled according to head differentials between boundary and neighboring cells; however, they are not explicitly calibrated. Calibration is accomplished indirectly by aiming for groundwater head levels within the watershed.

### Observational data gaps

The trend analysis was performed using calibrated model outputs at the subbasin scale (HUC12) instead of point-based observations (e.g., wells), due to significant gaps in observational data in the region that could create inaccuracies in direct trend studies. This regional-scale methodology offers significant insights but could be enhanced by subsequent research utilizing refined or more comprehensive observational datasets for validation.

## Summary and conclusions

In this study, we use the surface–subsurface hydrologic model, the Soil and Water Assessment Tool (SWAT+) augmented with new *gwflow* module for extensively irrigated region, Mississippi Alluvial Plain in United States. The SWAT + modeling code has been revised to incorporate irrigation applications and groundwater extraction for irrigation. This study connects cultivated areas allocated for groundwater irrigation to underlying aquifer grid cells, from which groundwater is taken according to irrigation demand, the efficiencies of selected irrigation methods (flood, sprinkler, drip), and the available groundwater storage. Soil moisture is used to trigger irrigation. This process simulates daily groundwater extraction for each agricultural land inside the model domain. The SWAT + model simulation spans the 2000–2020 period, including a two-year warm-up period (2000–2001), a seven-year calibration period (2002–2008), and a twelve-year testing period (2009–2020), using streamflow, groundwater head, and surface evapotranspiration data at sites throughout the river basin. Then the calibrated SWAT + model extended for period of 1982–2020 to investigate long-term of simulated groundwater head, groundwater evapotranspiration, groundwater pumping, recharge, and groundwater seepage. The linear quantile regression is used to perform trend analysis for both extremely wet and dry conditions as well as the average condition. From the results, we can conclude the following:

1. This study verifies the persistent depletion of groundwater storage in the Mississippi Alluvial Plain (MAP), predominantly caused by agricultural irrigation requirements, leading to a decrease in groundwater head in extensively irrigated subbasins with values of (–18.20 mm/yr.; wet condition), (–28.00 mm/yr.; dry condition), and (–20.60 mm/yr.; average condition).
2. A considerable decline trends are observed in groundwater evapotranspiration − 0.66 mm/yr. under wet conditions; − 0.38 mm/yr. under dry conditions; − 0.27 mm/yr. under average conditions), and simulated groundwater pumping (–9.96 mm/yr. under wet conditions; − 1.00 mm/yr. under dry conditions; − 1.12 mm/yr. under average conditions) in alluvial plains, especially in areas with extensive groundwater extraction for irrigation purposes. This underscores the significance of localized management solutions.
3. The alluvial plain exhibits significant increases for wet conditions in recharge rates, attaining up to 35.13 mm/yr., either due to irrigation return flow or precipitation events enhancing infiltration. Nonetheless, certain subbasins are showing reductions in recharge, as low as − 8.19 mm/yr. presumably because to diminishing water tables that constrain recharge capacity.
4. There is a decrease in recharge patterns for dry and average conditions, with values declining to − 5.51 mm/yr. (dry) and − 5.97 mm/yr. (average) in several subbasins. The declined recharge in several subbasins of the alluvial plain indicates a restricted ability for natural replenishment, potentially resulting from the depletion of groundwater levels or inadequate infiltration beneath irrigated farms.
5. Decreased groundwater evapotranspiration in extensively irrigated regions signifies that groundwater levels are approaching critical thresholds, constraining the aquifer’s natural discharge to sustain ecosystems and agricultural requirements.
6. The study recommends investigating artificial recharge methods and managed aquifer recharging (MAR) systems to augment groundwater storage and diminish reliance on pumping during peak irrigation times, due to the restricted natural recharge in the MAP.
7. The analysis indicates that diminishing groundwater seepage in irrigated regions intensifies store depletion, highlighting the necessity to monitor and manage seepage patterns as an integral component of groundwater conservation initiatives.

This study provides a new understanding of groundwater storage loss. This work highlights certain subbasin-level trends and sensitivities that have been missed in more comprehensive regional studies by considering fine-scale spatial and temporal variability. Furthermore, this work differs from previous studies that have mostly concentrated on groundwater pumping in that it integrates numerous hydrologic fluxes, enabling a thorough assessment of the factors causing storage loss. In addition to improving the precision of storage loss estimations, our method sheds light on the intricate relationships between groundwater usage and natural recharge processes, providing important direction for focused, sustainable management strategies in this heavily irrigated area.

**Table 1:** Features and model details of the 6 study watersheds.

HUC8 ^a^	Name	Area (km^2^)	NHD + Channels	HUC12 subbasins	HRUs ^b^	gwflow Cell Size (m)	gwflow Rows	gwflow Columns	gwflow Cells
8,030,202	Tallahatchie	2,723	1,556	30	7,031	500	218	143	31,174
8,030,204	Coldwater	4,820	3,005	54	8,117	500	183	231	42,273
8,030,205	Yalobusha	5,917	3,806	60	5,283	500	185	234	43,290
8,030,206	Upper Yazoo	4,330	2,514	51	6,626	500	220	154	33,880
8,030,207	Big Sunflower	8,210	4,279	87	30,082	500	421	176	74,096
8,030,209	Deer-Steele	2,121	883	30	5,939	500	253	71	17,963
Total	-	28,121	16,043	312	63,078	-	1,480	1,009	242,676

a: HUC 8 watershed is a hydrologic unit in the Watershed Boundary Dataset that is identified by an 8-digit Hydrologic Unit Code (HUC). These watersheds are delineated by USGS utilizing a nationwide system based on surface hydrologic characteristics.

b: Hydrologic response units (HRUs) are areas with similar distinctive characteristics of land use, soil, slope, and management within a subbasin.

**Table 2:** Datasets used to construct SWAT + models and the *gwflow* inputs.

Dataset		Resolution (m)	Source
SWAT + model	Land use, Land cover	30	U.S. Geological Survey, National Land Cover Data
	Field boundaries		Yan and Roy (2016)
	Topographic slope map	10	USGS National Elevation Dataset (Gesch et al., 2018)
	Weather		Global historical climatology network; PRISM
	Soil boundaries and properties	10	Soil Survey Staff (2014)
	Stream segments		NHD + Moore and Dewald (2016)
	Crop rotation		USDA–NASS, CDL
	Lakes and reservoirs		Moore and Dewald (2016)
	Water use		Dieter et al. (2018)
	Discharge from facilities		Skinner and Maupin (2019)
*gwflow*	Groundwater head	Vector Points	Bailey and Alderfer (2022)
	Aquifer thickness	250	Shangguan et al. (2017)
	Tile drainage	30	Valayamkunnath et al. (2020)
	Geologic units	Vector Polygons	Horton et al. (2017)

**Table 3:** Description and controlled hydrologic processes of the selected parameters with ranges for the SWAT + models for the study region.

Parameter	Parameter description	Hydrologic Processes	Lower Bound	Upper Bound	Scope
CN2 #	SCS runoff curve number for moisture condition II	Surface runoff processes	35	98	Land use – specific
slp_len #	Average slope length for erosion (m)	Lateral flow processes	10	150	HRUs – specific
esco #	Soil evaporation compensation factor	Potential and actual evapotranspiration processes	0.01	1	Global
epco #	Plant uptake compensation factor		0.01	1	
can_max #	Maximum canopy storage (mm H_2_O)		0.01	100	
rech_del #	Recharge delay (days)	Groundwater flow processes	1	30	Geologic zone – specific
syaqu #	Aquifer specific yield for a specific zone for i^th^ zone		0.05	0.35	
kaqu #	Aquifer hydraulic conductivity for a specific zone (m/day) for i^th^ zone		8.64E-06	15.00	
bed_thick	Streambed thickness (m)		0.10	1.00	
bed_k	Streambed hydraulic conductivity (m/day)		5E-08	1E-04	
bed_depth	River depth (m)		1.0	4.0	
tile_k	Hydraulic conductivity of the drain perimeter (m/day)		0.50	15.00	
tile_area	Area of groundwater inflow (m^2^) to tile		10.0	100.0	
tile_depth	Depth of tiles below ground surface (m)		1.00	2.00	
ch_n	Manning’s n for the main channels	Channel flow processes	0.01	0.30	Channel – specific
ch_k	Effective hydraulic conductivity of channels (mm/h)		0.01	500.00	
surq_lag	Surface runoff lag time (days)	Time of concentration	0.05	24	Global
awc #	Available water capacity of the soil layer (mm H_2_O/mm soil) for i^th^ layer	Soil water processes	0.01	1	Soil zone – specific
perco #	Percolation coefficient		0.01	1	

#: Signifies that multiple classes exist for the parameter. For example, rech_del values are assigned to various geologic units during parameters estimation.

**Table 4:** Monthly streamflow performance statistics for the SWAT + simulation in the MAP.

Watershed	Station	Calibration (2002–2008)				Testing (2009–2020)						
		NSE	*R* ^2^	PBIAS	KGE	NSE		*R* ^2^	PBIAS			KGE
Tallahatchie	USGS 07280400	0.84	0.84	–3.75	0.87	No observations						
Coldwater	USGS 07275900	0.91	0.92	–5.73	0.93	0.83		0.90	–27.8			0.71
Yalobusha	USGS 07281960	0.81	0.83	–15.50	0.80	0.85		0.89	–21.10			0.78
	USGS 07282090	0.70	0.76	19.80	0.61	0.74		0.77	18.40			0.69
	USGS 07281977	0.82	0.84	0.57	0.78	0.92		0.92	–5.58			0.89
	USGS 07283000	0.86	0.87	–13.30	0.84	0.80		0.86	–33.80			0.64
Upper Yazoo	USGS 07287160	0.87	0.87	–4.58	0.92	No observations						
	USGS 07287400	0.83	0.86	14.90	0.80	No observations						
Big Sunflower	USGS 07288500	0.49	0.65	–21.90	0.68	0.44		0.74	–27.90			0.55
	USGS 07288280	0.59	0.80	–24.40	0.61	0.76		0.89	–17.10			0.67

**Table 5:** Average annual hydrologic fluxes (mm) for each HUC8 within the LARB. The column headings are highlighted according to flux type. Bold = watershed inputs; italic = landscape and soil; bold italic = aquifer.

**Watershed**	**Precipitation**	Surface ET	*Surface Runoff*	*Lateral Flow*	*Tile Drain*	***Groundwater Seepage***	***GW Recharge***	***GW ET***	***Pumping***	*SW irrigation*
Tallahatchie	1474.4	898.8	667.0	11.3	0.0	6.1	52.51	1.1	13.7	23.5
Coldwater	1452.4	870.7	441.9	4.2	0.2	98.9	234.30	5.2	8.1	14.2
Yalobusha	1543.6	887.4	388.2	17.0	0.0	217.0	333.7	6.2	0.0	0.0
Upper Yazoo	1481.8	929.2	565.2	12.6	0.6	83.3	153.6	6.2	13.9	23.0
Big Sunflower	1451.2	1125.3	412.2	0.5	0.0	18.90	75.0	0.50	2.9	4.8
Deer-Steele	1482.0	955.3	709.9	2.9	0.0	11.7	43.5	1.3	3.0	5.1

## Electronic supplementary material

Below is the link to the electronic supplementary material.

Supplementary Material 1

## Data Availability

Data will be made available upon request (Salam Abbas, salam.a.abbas@colostate.edu).
