# Supplementary material for: Estimation of groundwater storage loss using surface–subsurface hydrologic modeling in an irrigated agricultural region
Source: Sci Rep. 2025 Mar 11;15:8350. doi: 10.1038/s41598-025-92987-6 (PMC11897132; doi:10.1038/s41598-025-92987-6)
Supplement: Supplementary file 1 — Supplementary Material 1 [file 41598_2025_92987_MOESM1_ESM.docx]

**Supporting Information: Estimation of Groundwater Storage Loss using Surface–Subsurface Hydrologic Modeling in an Irrigated Agricultural Region**

**Table S1.** Summary of statistically significant quantiles trends for different hydrologic variables for 6 watersheds in MAP.

| **HUC8 Watershed** | **Hydrologic Variable** | **Quantile** | **Number of Subbasins (HUC12) with Significant Trends** | **Range of p-values** |
| --- | --- | --- | --- | --- |
| **Tallahatchie** | Groundwater Head | 0.98 | 14 | 0.0000 – 0.0499 |
|  |  | 0.50 | 18 | 0.0000 – 0.0484 |
|  |  | 0.02 | 19 | 0.0000 – 0.0424 |
|  | Groundwater ET | 0.98 | 4 | 0.0001 – 0.0105 |
|  |  | 0.50 | 1 | 0.0230 |
|  |  | 0.02 | 1 | 0.0173 |
|  | Groundwater Pumping | 0.98 | 1 | 0.0498 |
|  |  | 0.50 | 6 | 0.0149 – 0.0499 |
|  |  | 0.02 | 1 | 0.0499 |
|  | Groundwater Recharge | 0.98 | 1 | 0.0498 |
|  |  | 0.50 | 0 | – |
|  |  | 0.02 | 0 | – |
|  | Groundwater Seepage | 0.98 | 11 | 0.0000 – 0.0238 |
|  |  | 0.50 | 8 | 0.0000 – 0.0450 |
|  |  | 0.02 | 3 | 0.0000 – 0.0239 |
| **Coldwater** | Groundwater Head | 0.98 | 6 | 0.0000 – 0.0388 |
|  |  | 0.50 | 11 | 0.0009 – 0.0458 |
|  |  | 0.02 | 3 | 0.0001 – 0.0494 |
|  | Groundwater ET | 0.98 | 0 | – |
|  |  | 0.50 | 1 | 0.0409 |
|  |  | 0.02 | 8 | 0.0011 – 0.0457 |
|  | Groundwater Pumping | 0.98 | 1 | 0.0147 |
|  |  | 0.50 | 0 | – |
|  |  | 0.02 | 4 | 0.0013 – 0.0199 |
|  | Groundwater Recharge | 0.98 | 5 | 0.0010 – 0.0415 |
|  |  | 0.50 | 1 | 0.0499 |
|  |  | 0.02 | 0 | – |
|  | Groundwater Seepage | 0.98 | 2 | 0.0049 – 0.0489 |
|  |  | 0.50 | 2 | 0.0283 – 0.0494 |
|  |  | 0.02 | 0 | – |

**Table S1.** Continued

| **HUC8 Watershed** | **Hydrologic Variable** | **Quantile** | **Number of Subbasins (HUC12) with Significant Trends** | **Range of p-values** |
| --- | --- | --- | --- | --- |
| **Yalobusha** | Groundwater Head | 0.98 | 6 | 0.0000 – 0.0285 |
|  |  | 0.50 | 16 | 0.0000 – 0.0342 |
|  |  | 0.02 | 49 | 0.0000 – 0.0478 |
|  | Groundwater ET | 0.98 | 9 | 0.0000 – 0.0412 |
|  |  | 0.50 | 6 | 0.0021 – 0.0312 |
|  |  | 0.02 | 9 | 0.0002 – 0.0237 |
|  | Groundwater Pumping | 0.98 | 0 | – |
|  |  | 0.50 | 0 | – |
|  |  | 0.02 | 0 | – |
|  | Groundwater Recharge | 0.98 | 2 | 0.0277 – 0.0388 |
|  |  | 0.50 | 0 | – |
|  |  | 0.02 | 11 | 0.0088 – 0.0499 |
|  | Groundwater Seepage | 0.98 | 7 | 0.0000 – 0.0471 |
|  |  | 0.50 | 6 | 0.0053 – 0.0421 |
|  |  | 0.02 | 16 | 0.0017 – 0.0499 |
| **Upper Yazoo** | Groundwater Head | 0.98 | 10 | 0.0000 |
|  |  | 0.50 | 22 | 0.0000 – 0.0396 |
|  |  | 0.02 | 20 | 0.0000 – 0.0411 |
|  | Groundwater ET | 0.98 | 7 | 0.0000 – 0.0324 |
|  |  | 0.50 | 5 | 0.0127 – 0.0429 |
|  |  | 0.02 | 12 | 0.0014 – 0.0440 |
|  | Groundwater Pumping | 0.98 | 0 | – |
|  |  | 0.50 | 2 | 0.0397 – 0.0499 |
|  |  | 0.02 | 2 | 0.0444 – 0.0498 |
|  | Groundwater Recharge | 0.98 | 2 | 0.0042 – 0.0234 |
|  |  | 0.50 | 0 | – |
|  |  | 0.02 | 0 | – |
|  | Groundwater Seepage | 0.98 | 11 | 0.0002 – 0.0458 |
|  |  | 0.50 | 7 | 0.0000 – 0.0463 |
|  |  | 0.02 | 8 | 0.0003 – 0.0485 |

**Table S1.** Continued

| **HUC8 Watershed** | **Hydrologic Variable** | **Quantile** | **Number of Subbasins (HUC12) with Significant Trends** | **Range of p-values** |
| --- | --- | --- | --- | --- |
| **Big Sunflower** | Groundwater Head | 0.98 | 38 | 0.0000 – 0.0426 |
|  |  | 0.50 | 29 | 0.0000 – 0.0484 |
|  |  | 0.02 | 36 | 0.0000 – 0.0411 |
|  | Groundwater ET | 0.98 | 28 | 0.0000 – 0.0440 |
|  |  | 0.50 | 28 | 0.0000 – 0.0480 |
|  |  | 0.02 | 1 | 0.0004 |
|  | Groundwater Pumping | 0.98 | 13 | 0.0000 – 0.0433 |
|  |  | 0.50 | 3 | 0.0193 – 0.0286 |
|  |  | 0.02 | 0 | – |
|  | Groundwater Recharge | 0.98 | 22 | 0.0009 – 0.0489 |
|  |  | 0.50 | 0 | – |
|  |  | 0.02 | 0 | – |
|  | Groundwater Seepage | 0.98 | 19 | 0.0000 – 0.0499 |
|  |  | 0.50 | 6 | 0.0001 – 0.0493 |
|  |  | 0.02 | 14 | 0.0000 – 0.0399 |
| **Deer-Steele** | Groundwater Head | 0.98 | 11 | 0.0000 – 0.0279 |
|  |  | 0.50 | 5 | 0.0000 – 0.0010 |
|  |  | 0.02 | 6 | 0.0000 – 0.0043 |
|  | Groundwater ET | 0.98 | 4 | 0.0110 – 0.0395 |
|  |  | 0.50 | 3 | 0.0000 – 0.0288 |
|  |  | 0.02 | 0 | – |
|  | Groundwater Pumping | 0.98 | 0 | – |
|  |  | 0.50 | 3 | 0.0000 – 0.0434 |
|  |  | 0.02 | 0 | – |
|  | Groundwater Recharge | 0.98 | 1 | 0.0481 |
|  |  | 0.50 | 0 | – |
|  |  | 0.02 | 0 | – |
|  | Groundwater Seepage | 0.98 | 10 | 0.0000 – 0.0497 |
|  |  | 0.50 | 4 | 0.0000 – 0.0024 |
|  |  | 0.02 | 6 | 0.0000 – 0.0391 |

**Model Data References**

Bailey, R., and Alderfer, C. (2022). Groundwater Data in Unconfined Aquifers - conterminous United States. *figshare*. Collection. <https://doi.org/10.6084/m9.figshare.c.5918738.v2>

Dieter, C., Maupin, M., Caldwell, R., Harris, M., Ivahnenko, T., Lovelace, J., Barber, N., and Linsey, K. (2018). *Water availability and use science program: Estimated use of water in the United States in 2015* (Circular 1441). US Geological Survey. <https://doi.org/10.3133/cir1441>

Gesch, D., Evans, G., Oimoen, M., and Arundel, S. (2018). The national elevation dataset (pp. 83–110). American Society for Photogrammetry and Remote Sensing.

Horton, J., San Juan, C., and Stoeser, D. (2017). *The state geologic map compilation (SGMC) geodatabase of the conterminous United States, ver. 1.1, August 2017* (Data Series 1052). <https://doi.org/10.3133/ds1052>

Moore, R., and Dewald, T. (2016). The road to nhdp lus — advancements in digital stream networks and associated catchments. *J. Am. Water Resour. As.*, 52(4), 890-900. <https://doi.org/10.1111/1752-1688.12389>

Shangguan, W., Hengl, T., Mendes de Jesus, J., Yuan, H., and Dai, Y. (2017). Mapping the global depth to bedrock for land surface modeling. *J. Adv. Model. Earth Syst.*, 9(1), 65-88. <https://doi.org/10.1002/2016ms000686>

Skinner, K., and Maupin, M. (2019). *Point-source nutrient loads to streams of the conterminous United States, 2012* (No. 1101). US Geological Survey. <https://doi.org/10.3133/ds1101>

Soil Survey Staff (2014). Gridded soil survey geographic (gSSURGO) database for the conterminous United States.

Valayamkunnath, P., Barlage, M., Chen, F., Gochis, D., and Franz, K. (2020). Mapping of 30-meter resolution tile-drained croplands using a geospatial modeling approach. *Sci. Data*, *7*(1), 257. <https://doi.org/10.1038/s41597-020-00596-x>

Yan, L., and Roy, D. (2016). Conterminous United States crop field size quantification from multi-temporal Landsat Data. *Remote Sensing of Environment*, 172, 67-86 <https://doi.org/10.1016/j.rse.2015.10.034>
